# *Mycobacterium tuberculosis* Type VII Secreted Effector EsxH Targets Host ESCRT to Impair Trafficking

**DOI:** 10.1371/journal.ppat.1003734

**Published:** 2013-10-31

**Authors:** Alka Mehra, Aleena Zahra, Victor Thompson, Natalie Sirisaengtaksin, Ashley Wells, Maura Porto, Stefan Köster, Kristen Penberthy, Yoshihisha Kubota, Amelie Dricot, Daniel Rogan, Marc Vidal, David E. Hill, Andrew J. Bean, Jennifer A. Philips

**Affiliations:** 1 Division of Infectious Diseases, Department of Medicine, Department of Pathology and Department of Microbiology, New York University School of Medicine, New York, New York, United States of America; 2 Department of Neurobiology and Anatomy, and Graduate School of Biomedical Sciences, The University of Texas Health Science Center at Houston, Houston, Texas, United States of America; 3 Center for Cancer Systems Biology (CCSB) and Department of Cancer Biology, Dana-Farber Cancer Institute, Boston, Massachusetts, United States of America; 4 Department of Genetics, Harvard Medical School, Boston, Massachusetts, United States of America; 5 Division of Pediatrics, The University of Texas M.D. Anderson Cancer Center, Houston, Texas, United States of America; University of Massachusetts, United States of America

## Abstract

*Mycobacterium tuberculosis* (Mtb) disrupts anti-microbial pathways of macrophages, cells that normally kill bacteria. Over 40 years ago, D'Arcy Hart showed that Mtb avoids delivery to lysosomes, but the molecular mechanisms that allow Mtb to elude lysosomal degradation are poorly understood. Specialized secretion systems are often used by bacterial pathogens to translocate effectors that target the host, and Mtb encodes type VII secretion systems (TSSSs) that enable mycobacteria to secrete proteins across their complex cell envelope; however, their cellular targets are unknown. Here, we describe a systematic strategy to identify bacterial virulence factors by looking for interactions between the Mtb secretome and host proteins using a high throughput, high stringency, yeast two-hybrid (Y2H) platform. Using this approach we identified an interaction between EsxH, which is secreted by the Esx-3 TSSS, and human hepatocyte growth factor-regulated tyrosine kinase substrate (Hgs/Hrs), a component of the endosomal sorting complex required for transport (ESCRT). ESCRT has a well-described role in directing proteins destined for lysosomal degradation into intraluminal vesicles (ILVs) of multivesicular bodies (MVBs), ensuring degradation of the sorted cargo upon MVB-lysosome fusion. Here, we show that ESCRT is required to deliver Mtb to the lysosome and to restrict intracellular bacterial growth. Further, EsxH, in complex with EsxG, disrupts ESCRT function and impairs phagosome maturation. Thus, we demonstrate a role for a TSSS and the host ESCRT machinery in one of the central features of tuberculosis pathogenesis.

## Author Summary

*Mycobacterium tuberculosis* (Mtb) causes the disease tuberculosis, one of the world's most deadly infections. The host immune system can't eradicate Mtb because it grows within macrophages, cells that normally kill bacteria. One of the intracellular survival strategies of Mtb is to avoid delivery to lysosomes, a phenomenon described over 40 years ago, but for which the mechanism and molecular details remain incomplete. Mtb possess specialized secretion systems (Type VII secretion systems; TSSS) that transfer particular proteins out of the bacteria, but how these proteins promote infection is not well understood. In this study, we used a high stringency yeast two-hybrid system to identify interactions between secreted effectors from Mtb and human host factors. We identified ninety-nine such interactions and focused our attention on the interaction between EsxH, secreted by Esx-3, a TSSS of Mtb, and Hrs, a component of the host ESCRT machinery. We provide evidence that Mtb EsxH directly targets host Hrs to disrupt delivery of bacteria to lysosomes. Thus, this study demonstrates the role of a TSSS effector and the ESCRT machinery in what is one of the central features of tuberculosis pathogenesis, thereby providing molecular insight into why humans can't clear Mtb infection.

## Introduction

An important virulence property of *Mycobacterium tuberculosis* (Mtb)- the causative agent of the disease tuberculosis- is its ability to avoid delivery to the lysosome. It has long been appreciated that Mtb alters phagosome maturation, such that internalized bacteria are not transported to the lysosome but instead reside in an early endosome-like compartment [1], [2]. The Mtb-induced block in phagosome-lysosome fusion has been attributed to a wide array of lipid and protein effectors [3], [4] but the mechanism remains poorly understood. More recently, the ability of Mtb to permeabilize the phagosomal membrane, which allows bacterial products and in some cases intact bacteria to access the cytosol, has been described [5]–[9]. The TSSS Esx-1 and its secreted effectors, EsxA/ESAT-6 and EsxB/CFP-10, are critical for this process. Esx-1 has been investigated intensively because its absence in the vaccine strain *Mycobacterium bovis*-BCG (BCG) largely accounts for attenuation of that strain [8]–[10]. Mtb encodes five loci resembling Esx-1 (Esx-1-Esx-5), as well as 11 tandem pairs of proteins similar to EsxA and EsxB (EsxA-EsxW), but their cellular targets, if any, are unknown [11]. Esx-3 plays a role in iron acquisition in Mtb, as well as in a non-pathogenic strain, *Mycobacterium smegmatis* (Msmeg) [12], [13]. Esx-3 is a focus of vaccine efforts because it secretes EsxG/TB9.8 and EsxH/TB10.4, which are highly antigenic [14], [15], and because introduction of the Mtb ESX-3 locus into an Msmeg strain lacking the endogenous ESX-3 region generates highly protective immunity [16]. The ESX-5 locus is required for transport of proteins with conserved proline-glutamic acid (PE) and proline-proline-glutamic acid (PPE) motifs [17], [18] and modulates macrophage responses [19]. Thus, TSSSs and their putative effectors appear to be important in virulence and modulating host cells, however, their mechanism of action and molecular targets are unclear.

Here, we show that EsxG and EsxH from Mtb, but not the Msmeg homologs, target the host factor, Hrs. Hrs is a component of the ESCRT machinery, a group of four protein complexes (ESCRT-0 to ESCRT-III) composed of cytosolic components that are sequentially recruited to the endosomal membrane. The ESCRT machinery has a well-described role in directing cargo destined for degradation into intraluminal vesicles of multivesicular bodies (MVBs) that fuse with lysosomes [20], [21]. We show that ESCRT is also required to deliver Mtb to the lysosome and to restrict intracellular bacterial growth. EsxH, in complex with EsxG, is able to disrupt ESCRT function and impair phagosome maturation.

## Results

### High throughput identification of Mtb secretome-human host interactions

We used a systematic strategy to identify secreted bacterial virulence factors by looking for interactions with host proteins using a high throughput, high stringency, yeast two-hybrid (Y2H) platform [22]. First, we curated the literature to define the Mtb secretome. Thirty-eight publications predicted 718 secreted proteins based on presence in culture filtrate (CF), ability to cause secretion of an assayable protein, bioinformatic criteria, or detailed study (see Text S1 for additional details). In order to prioritize open reading frames (ORFs) for screening, we imposed a number of criteria, such as excluding proteins with multiple transmembrane spanning domains (see Text S1 for additional details). In addition, since the starting list of putative secreted proteins might contain proteins that are not actually secreted, we attempted to eliminate ORFs that were likely to be inaccurately classified as secreted. One way in which this can happen is if cytoplasmic proteins appear in CF due to bacterial lysis. In order to minimize the contribution of such proteins, we did not include ORFs that were annotated in Tuberculist (http://tuberculist.epfl.ch/) as being involved in lipid metabolism, information pathways (which contains proteins involved in replication, transcription and translation), or intermediary metabolism and respiration, since most of these are likely involved in basic, intrinsic bacterial processes, and hence, many may be misclassified. To avoid removing true secreted proteins, ORFs were not de-prioritized if they had a possible signal sequence or there were data supporting their role during infection. In doing so, we removed many proteins that were found in CF in a single study, and hence may be misclassified (see Text S1 for details). From the final list, 339 sequence validated secretome ORFs were provided by Pathogen Functional Genomic Resource Center (PFGRC; Dataset S1). Because many secreted proteins play an intrinsic role in the bacterial lifecycle, we anticipated that only a small fraction would interact with human proteins. Thus, to estimate a false positive hit rate of our system, we included sixty ORFs that are not likely to be secreted to serve as controls (see Text S1 for details; Dataset S2).

In order to evaluate their performance in Y2H protein-protein interaction (PPI) mapping, we tested the 399 Mtb ORFs expressed as Gal4-DNA binding domain (DB) fusions for pair wise interactions with the same 399 Mtb ORFs expressed as Gal4-activation domain (AD) fusions. From the ∼160,000 combinations queried, we found 14 unique PPIs (Table S1). The rate of interactions is as high as in human ORFeome mapping [22], exceeding the stochastic false positive rate of the Y2H platform by fifteen-fold [23]. Half of the interactions were between proteins belonging to the WXG100 family (EsxA-EsxW). These proteins are approximately 100 amino acids in length and have a characteristic hairpin bend formed by a Trp-Xaa-Gly (W-X-G) motif. Mtb encodes 11 tandem pairs of such proteins, which are thought to function as secreted heterodimers. Heterodimer formation is proposed to be limited to interactions between genome pairs or very closely related family members [24], [25], and the interactions we detected by Y2H exhibit this specificity. Six of the remaining seven PPIs involved homotypic interactions; for example, bacterioferritin (BfrB) was found to interact with itself, consistent with the proposal that it assembles into 24-subunit oligomers [26].

After ensuring the high performance of Mtb ORFs in Y2H PPI mapping, we looked for interactions between the Mtb secretome and ∼12,000 human ORFs, testing approximately 4 million interactions. From the secretome collection, we identified 99 PPIs between 53 Mtb proteins and 63 human proteins (Dataset S3). The number of Mtb proteins exhibiting an interaction with a human protein was approximately two-fold higher for the secretome collection compared to the non-secreted control set (53 out of 339 versus 5 out of 60). We analyzed the collection to determine whether PPIs were enriched for subsets of Mtb proteins (Table S2). We observed that the sixteen Esx proteins included in the collection were significantly more likely to interact with human ORFs than were controls (p = 0.0087). The finding that Esx proteins were enriched for interactions may reflect that this group of proteins plays an important role in virulence, or could mean that these proteins, which usually form a heterodimer, are prone to aberrant interactions when they are expressed without their binding partner.

It is difficult to gauge the success of the screen based upon known interaction between Mtb proteins and cytosolic human protein because so few are known. Included in our screening set were, PtpA, which has been shown to interact with Vps33B and the H subunit of the human v-ATPase [27], [28], LpdC, which interacts with coronin 1 [29], and NdkA, which interacts with Rab5 and Rab7 [30]. We did not identify these known interactions, however, the screen was not performed to saturation and the Y2H platform can detect ∼20% of well-validated interacting pairs [31]. We did identify an interaction between PtpA and Ligand of Numb protein X (LNX1), a RING finger-type E3 ligase that contains 4 PDZ domains and plays a scaffolding role in diverse cellular pathways. Several other secretome ORFs also interacted with LNX1, suggesting that LNX1 might be modulated by Mtb, or LNX1 might regulate the function or stability of certain Mtb effectors.

When we examined hit rates based upon the functional category of the Mtb ORFs, the category with the greatest enrichment was cell wall and cell processes, which contains the Esx family members. The intermediary metabolism and respiration category exhibited a hit rate similar to the control collection, consistent with the idea that most of these ORFs do not function in host interactions as their annotation suggests. Interestingly, one of the two proteins that did exhibit an interaction in this category is Zmp1, a zinc metalloprotease which has been shown to inhibit the inflammasome and impair phagosome maturation but whose cellular target is unknown [32]. Zmp1 interacted with KCTD6, a BTB/POZ domain containing protein that can function as a Cullin3 (Cul3) adaptor [33]. Cul3 has recently been shown to be a regulator of endo-lysosomal trafficking, suggesting that Zmp1 may impair phagosome maturation by acting on KCTD6-Cul3 [34].

To evaluate the human targets of the Mtb proteins, we searched the STRING database (http://string.embl.de/) for each of the proteins' functional associations. The STRING database predicts protein-protein interactions based upon physical and functional associations, such as available high-throughput data, co-expression, genomic context, and text mining of available literature. Using a medium confidence value to define protein-protein interactions, there were significantly more interactions (n = 16) observed for the human targets than would be predicted by chance (p = 8.3×10^−12^). We identified proteins involved in host immunity to bacterial infection, such as Ndp52 [35], [36], Tax1pb1 [37], and STAT3 [38], however, human targets of the Mtb proteins were not significantly enriched for annotated pathways in the Kyoto Encyclopedia of Genes and Genomes (KEGG) when corrected for multiple testing, which may reflect the limited number of human targets found.

### ESCRT is required for restricting intracellular growth and trafficking of slow growing mycobacteria

**Figure 1:**
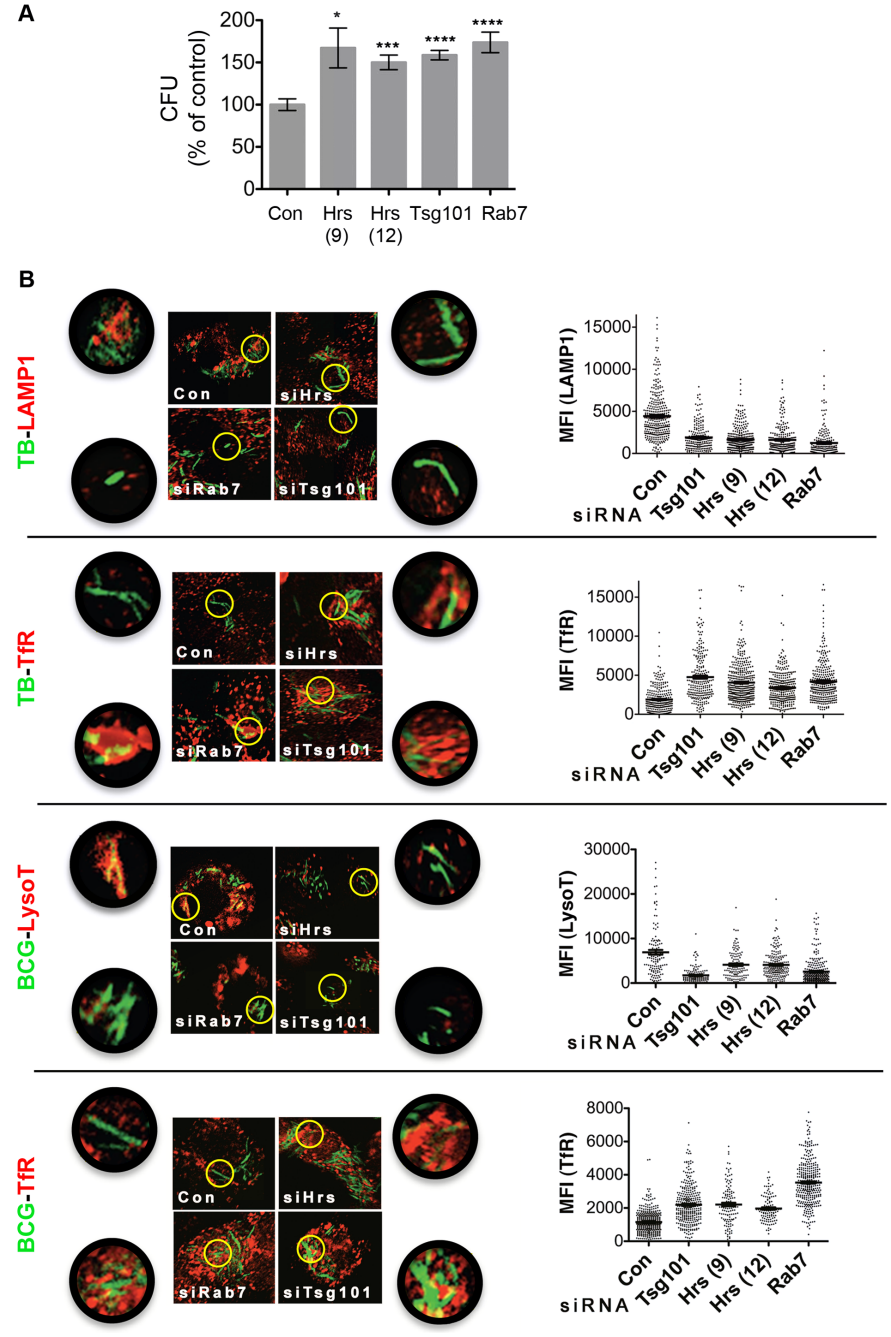
ESCRT is required to traffic Mtb to the lysosome. (A) RAW264.7 cells were treated with control siRNA (Con), individual siRNAs targeting Hrs (#9 or #12), or siRNA pools targeting Tsg101 or Rab7 and infected with Mtb. Bacterial colony forming units (CFU) were enumerated 2 d post-infection and are normalized to the average number of CFU in control wells from three independent experiments. Results reflect the mean +/− SEM. *p = 0.018; ***p = 0.0002; ****p<0.0001, unpaired Student's *t*-test. (B) Composite images and quantification of Mtb-GFP or BCG-GFP (in green) and RAW cell LAMP1, TfR, or LysoTracker (in red) at 24 hpi. Regions indicated by yellow circles are shown in higher magnification in adjacent panels. In graphs, data points are the mean fluorescence intensity (MFI) around at least 100 phagosomes for each condition; bars show mean +/− SEM. Data are representative of at least three experiments; *p*<0.0001 for all siRNAs compared to controls.

We focused on the interaction between EsxH and Hrs because TSSSs, which secrete Esx proteins, are clearly important in virulence but the function of their secreted effectors is largely unknown. In addition, our existing data supported the idea that the ESCRT machinery is important in controlling bacterial replication. Hrs, which plays a central role in the assembly of the initial ESCRT components on endosomes, is recruited to mycobacterial phagosomes [39], and we had previously shown in an RNAi screen in *Drosophila* that ESCRT restricts the intracellular growth of rapidly growing mycobacteria [40], [41]. Control of bacterial replication appears to be particularly sensitive to ESCRT perturbation, because, in addition, when we screened ∼6500 siRNA pools in RAW 264.7 (RAW) macrophages for their ability to confer enhanced intracellular growth of Msmeg, we found that the two strongest hits were Rab7, known to be involved in late endosome-lysosome fusion, and Tsg101, an ESCRT-I component that is recruited to endosomes by Hrs (data not shown). Hrs was also identified in this screen, although previously we had found no effect with Hrs silencing, which we now attribute to insufficient protein depletion [41]. In the RAW cell RNAi screen that identified Hrs, we used Ambion Silencer siRNA pools, whereas previously we used a Dharmacon siGENOME pool to deplete Hrs [41]. To clarify the discrepancy, we tested a third pool (Dharmacon ON-TARGET*plus*), which, like the Ambion pool, conferred enhanced growth to Msmeg. We tested the individual Dharmacon ON-TARGET*plus* siRNAs and found that 2 of 4 targeting Hrs resulted in depletion of Hrs protein, enhanced the growth of Msmeg, and altered trafficking, whereas the other two had no effect (Figure S1 and data not shown). Thus, one possibility is that Mtb secretes EsxH, which binds Hrs and impairs ESCRT function, thereby promoting intracellular bacterial growth. To determine whether ESCRT restricts growth of Mtb, we depleted Hrs and Tsg101 and examined the intracellular growth of Mtb in RAW macrophages. We found no significant effect of silencing on bacterial uptake (data not shown), however when we assessed bacterial colony forming units (CFU) two day post-infection, we observed enhanced intracellular survival of Mtb in cells depleted of Hrs or Tsg101, similar to what was seen with Rab7 silencing (Figure 1A). Intracellular growth of BCG in bone marrow-derived macrophages (BMDMs) was even more strongly effected (Figure S2). Thus, Hrs restricts growth of slow growing and virulent mycobacteria.

ESCRT targets certain cell surface receptors and biosynthetic cargo to lysosomes [42]. Thus, ESCRT might restrict intracellular bacterial growth by governing bacterial trafficking and/or lysosomal content. We examined the localization of Mtb relative to Transferrin Receptor (TfR), a marker of early and recycling endosomes, and LAMP1, a marker of late endosomes and lysosomes using automated image analysis (Figures S3A, S3B). In cells depleted of Tsg101, Hrs, or Rab7, we observed diminished co-localization between Mtb and LAMP1 and a concomitant increase in co-localization of Mtb with TfR compared to control cells 24 hours post-infection (hpi) (Figure 1B), suggesting decreased Mtb delivery to degradative compartments. Similarly, in cells infected with BCG there was diminished co-localization with LysoTracker, which accumulates in the acidic environment of the lysosome, and enhanced co-localization with TfR (Figure 1B). Thus, Hrs and Tsg101, like Rab7, are required for bacterial trafficking. To verify that bacterial viability correlates with low LAMP1 and LysoTracker co-localization and with high TfR co-localization, we compared the cellular localization of viable bacteria to total bacteria. We identified metabolically active BCG 24 hpi based upon their ability to induce expression of GFP from a tetracycline-inducible promoter (BCG-tet-GFP) and compared their intracellular localization to the BCG strain that constitutively express GFP (BCG-GFP). Whereas there was a wide range in intensities of associated LAMP1 and LysoTracker with BCG-GFP, metabolically active bacteria were found almost exclusively in phagosomes with minimal acidification, little co-localization with LAMP1, and enhanced TfR co-localization at 48 hpi (Figure S3C). Thus, impaired bacterial trafficking to a late endosomal or lysosomal compartment underlies the failure to control mycobacterial replication in ESCRT-depleted cells, although altered lysosomal content may also contribute.

### The Mtb EsxG EsxH heterodimer binds Hrs

**Figure 2:**
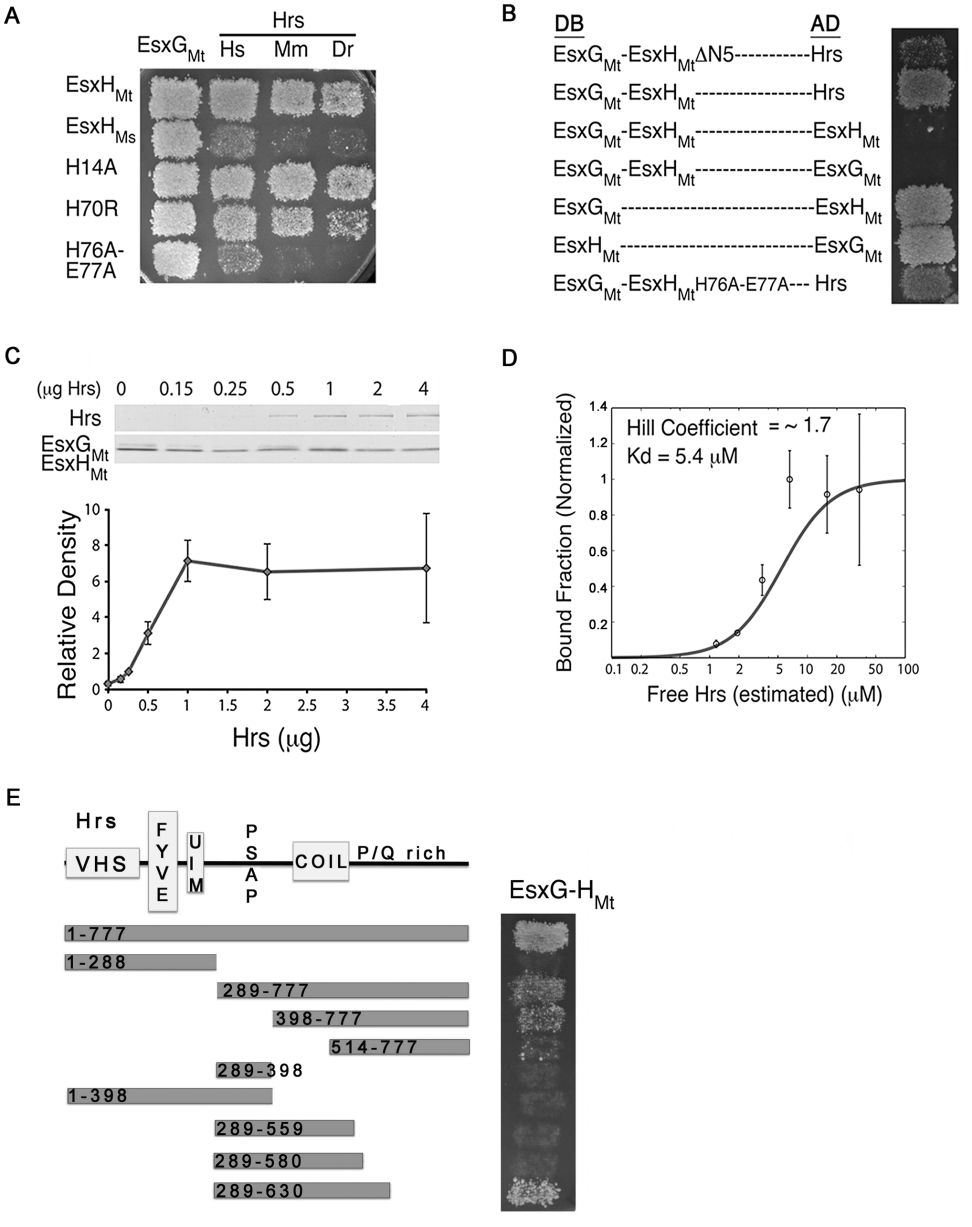
EsxH_Mt_ binds Hrs. (A) Gal4 DNA-binding domain (DB) fusions of EsxH_Mt_, EsxH_Ms_, or mutant EsxH_Mt_ were tested for Y2H interactions with Gal4 activation-domain (AD) fusions of EsxG_Mt_, human (Hs), mouse (Mm), or zebrafish (Dr) Hrs. (B) Y2H interaction between indicated DB and AD constructs. Hrs is human. EsxG_Mt_-EsxH_Mt_-DB did not interact with EsxG_Mt_-AD or EsxH_Mt_-AD, presumably because of the intramolecular interaction in the DB construct. (C) Increasing amounts of Hrs were incubated with a constant amount of immobilized EsxG_Mt_-EsxH_Mt_ and bound fraction examined by Coomassie blue. (D) Average binding (n = 3) was fitted with the Hill function, revealing a Hill coefficient of ∼1.7 and a K_D_ of 5.4 µM. (E) EsxG_Mt_-EsxH_Mt_-DB was tested in the Y2H for interactions with human Hrs-AD deletion constructs. The domain structure of Hrs is indicated.

Pathogenic mycobacteria arrest phagosome maturation in evolutionarily diverse cells. Supporting the idea that EsxH might play a role in inhibition of bacterial degradation, we observed that EsxH interacts with human, mouse, and zebrafish orthologs of Hrs, suggesting that it recognizes a conserved structural feature of Hrs (Figure 2A). Orthologs of EsxH are found widely in mycobacteria, including in the non-pathogen Msmeg. If EsxH prevents phagosome-lysosome fusion by impairing Hrs function, we anticipate that would be a feature specific to EsxH from pathogenic mycobacteria. To test this, we cloned the EsxH ortholog from Msmeg (MSMEG_0621; EsxH_Ms_), which encodes a protein 75% identical to EsxH from Mtb (hereafter referred to as EsxH_Mt_). Although EsxH_Ms_ interacted with EsxG_Mt_, demonstrating that the protein was functional in the Y2H assay, it interacted poorly with Hrs (Figure 2A), consistent with the notion that the interaction of EsxH_Mt_ with Hrs contributes to virulence.

EsxH_Mt_ forms a heterodimer with EsxG_Mt_, composed of a four-helix bundle with flexible N- and C-terminal arms from both proteins that coordinate zinc and contribute to a cleft that has been predicted to mediate a PPI [43]. To determine whether Hrs interacts with the heterodimer, we used a fusion protein in which EsxG_Mt_ and EsxH_Mt_ were expressed as a single polypeptide that preserves the folded structure of the native heterodimer [44]. This fusion protein interacted with Hrs (Figure 2B). Deletion of the first five amino acids of EsxH_Mt_ (EsxG_Mt_-EsxH_Mt_-ΔN5) weakened its interaction with Hrs (Figure 2B). These data show that Hrs can interact with EsxH_Mt_ when it is complexed to EsxG_Mt_ and suggest that the conformation of the amino terminal arm of EsxH_Mt_ is important. To further test whether the structure of the N- and C-termini are important, we mutated His-14, His-70, and His-76 Glu-77. These residues contribute to zinc binding, and His-76 is also part of the predicted cleft. We mutated them to Ala, with the exception of His-70, which we changed to Arg because this is found in EsxH_Ms_. While H14A and H70R did not have a detectable effect, when His-76 and Glu-77 were both changed to Ala, the interaction between EsxH_Mt_ and Hrs was impaired, although EsxH_Mt_ H76A-E77A still interacted with EsxG_Mt_ (Figure 2A). To verify that EsxH_Mt_ binds Hrs, we purified the EsxG_Mt_ EsxH_Mt_ heterodimer from *E. coli* [44] and Hrs from baculovirus [45]. Hrs bound EsxG_Mt_ EsxH_Mt_ in a saturable manner, exhibiting stoichiometric binding with a Kd of ∼5 µM (Figure 2C and 2D). We conclude that Hrs interacts with the EsxG_Mt_ EsxH_Mt_ heterodimer, and the interaction likely involves the N- and C-terminal arms of EsxH_Mt_.

### EsxG_Mt_ and EsxH_Mt_ disrupt ESCRT function in mammalian cells

**Figure 3:**
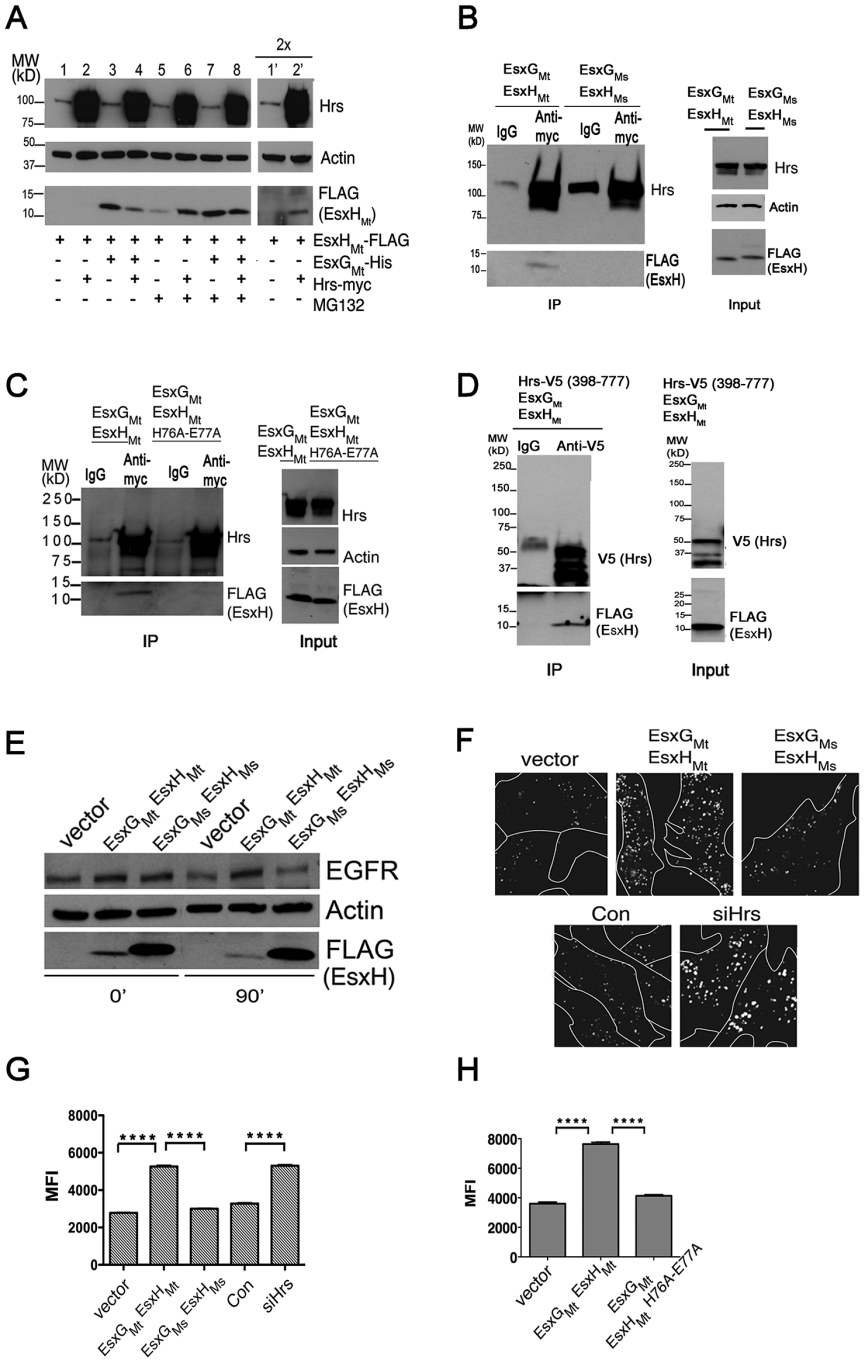
EsxG_Mt_ and EsxH_Mt_ interact with Hrs and disrupt ESCRT function in mammalian cells. (A) EsxH_Mt_-FLAG, EsxG_Mt_-His, and Hrs-myc expressed in HEK293 cells. DMSO or MG132 were added 3 h prior to protein harvest and samples were analyzed by western blotting. Lanes 1′ and 2′ are identical to 1 and 2 except that twice the amount of protein was loaded. Quantification from three independent experiments is shown in Figure S4. (B) Immunoprecipitation (IP) of Hrs using antibody recognizing myc tag or isotype control from HEK293 cells expressing Hrs-myc and either EsxG_Mt_-His EsxH_Mt_-FLAG or EsxG_Ms_-His EsxH_Ms_-FLAG. MG132 was used as pre-treatment. Western blot of IP and input were probed with antibodies as indicated. (C) IP of Hrs-myc in HEK293 cells with antibody recognizing myc tag or isotype control from HEK293 cells expressing Hrs-myc, EsxG_Mt_-His, and either EsxH_Mt_-FLAG or EsxH_Mt_-H76A-E77A-FLAG. MG132 was used as pre-treatment. Western blot of IP and input were probed with antibodies as indicated. (D) IP of C-terminal fragment of Hrs (amino acids 398–777) using antibody recognizing V5-tag or isotype control from HEK293 cells expressing Hrs-398–777-V5, EsxG_Mt_-His, EsxH_Mt_-FLAG. MG132 was used as pre-treatment. Western blot of IP and input were probed with antibodies as indicated. (E) HEK293 cells transfected with indicated plasmids were incubated with EGF for 0 or 90 min prior to western analysis. (F–H) A549 cells transfected with plasmids or siRNAs were imaged 90 min after incubation with Alexa-488 EGF. In F, white lines indicate cell borders. (G) and (H), MFI of at least 800 endosomes from at least 30 cells. Black bars show mean +/− SEM. ****p<0.0001 between indicated conditions, unpaired Student's *t*-test. No MG132 was used in experiments E–H. Data are representative of at least three independent experiments.

To determine whether EsxH_Mt_ interacts with Hrs and alters ESCRT function in mammalian cells, we expressed EsxH_Mt_–FLAG in HEK293 cells. EsxH_Mt_ was not detectable unless we co-expressed EsxG_Mt_. (Figure 3A, compare lanes 1 and 3; see Figure S4 for quantification); its abundance was also increased slightly by overexpression of Hrs (compare lane 1′ with 2′ and lane 5 with 6). When expressed alone, EsxH_Mt_ could be stabilized by MG132, likely because it is not properly folded without EsxG_Mt_ and hence is subject to proteasome-mediated degradation (Figure 3A compare lanes 1 and 5). To determine if there was an interaction between EsxG_Mt_-EsxH_Mt_ and Hrs, we performed co-immunoprecipitation experiments in cells co-transfected with EsxG_Mt_, EsxH_Mt_, and Hrs-myc. Hrs was immunoprecipitated with an antibody directed against the myc-tag, and we found that EsxH_Mt_ was co-immunoprecipitated (Figure 3B). No EsxH_Mt_ was co-immunoprecipitated when an isotype control antibody was used, and as expected, EsxH_Ms_ and EsxH_Mt_-H76A E77A were impaired in the interaction with Hrs (Figure 3B and 3C). Interestingly, the co-immunoprecipitation of EsxH_Mt_ and Hrs could only be detected when cells were pre-treated with MG132. Thus, one possibility is that the EsxG_Mt_-EsxH_Mt_ heterodimer is polyubiquitinated and degraded by the proteasome. In the presence of MG132, the polyubiquitinated species might accumulate, allowing us to detect an interaction between Hrs and an ubiquitinated species of EsxH_Mt_, since Hrs contains an ubiquitin interacting motif (UIM) domain. Arguing against this possibility, when EsxG_Mt_ was co-expressed with EsxH_Mt_, there was little, if any, effect of MG132 on EsxH_Mt_ protein levels (Figure 3A, compare lanes 3 and 7, Figure S4, and Figure S5). In addition, when we examined mono- and polyubiquitinated proteins using the FK2 antibody, inhibition of the proteasome with MG132 caused the accumulation of high molecular weight proteins as anticipated. However, there was no difference seen in the quantity or mobility of EsxH_Mt_ (Figure S5). Further, when we mapped the region of Hrs required for the interaction with EsxG_Mt_-EsxH_Mt_ in the Y2H assay, the UIM domain was not required. Amino acids 398–630, which contain a coiled-coil region, were sufficient to mediate the interaction (Figure 2E). We verified that the C-terminal half of Hrs was sufficient to mediate an interaction by co-immunoprecipitation (Fig. 3D). In summary, these data show that EsxG_Mt_ stabilizes EsxH_Mt_ in the mammalian cytosol and that the heterodimer can bind the C-terminus of Hrs.

To test the hypothesis that the EsxH_Mt_ interaction with Hrs disrupts ESCRT, we examined the effect of EsxG_Mt_ EsxH_Mt_ on epidermal growth factor (EGF) and epidermal growth factor receptor (EGFR) degradation (all performed without MG132 treatment). Upon binding ligand, EGFR is internalized and transferred into ILVs by ESCRT so that it can be degraded upon MVB-lysosome fusion. To determine whether EsxH_Mt_ interferes with this process, we transfected EsxG_Mt_ and EsxH_Mt_ or vector control into HEK293 cells and examined EGFR levels 90 min after EGF treatment. We found that EGFR levels decreased in control cells. In cells co-expressing EsxG_Mt_ and EsxH_Mt_ there was a 63+/−8% (n = 3) increase in the fraction of EGFR that remained undegraded (Figure 3E), similar to what has been seen with Hrs depletion [46]. In contrast, co-expression of EsxG_Ms_ and EsxH_Ms_ had no detectable effect on EGFR degradation (Figure 3E). We observed similar results when we used fluorescent EGF to examine trafficking in A549 cells using fluorescence microscopy. As expected, cells depleted of Hrs showed enhanced EGF fluorescence due to impaired degradation. We observed a similar decreased degradation in cells expressing EsxG_Mt_ and EsxH_Mt_ (Figure 3F and G). In contrast, expression of EsxG_Ms_ EsxH_Ms_ or EsxG_Mt_ EsxH_Mt_-H76A-E77A had little effect on EGF degradation (Figure 3G and 3H). Thus, EsxH_Mt_, in complex with EsxG_Mt_, is sufficient to inhibit EGF and EGFR degradation, an activity that correlates with its binding to Hrs. EsxH_Ms_, from the non-pathogenic species, does not have this property.

### EsxG_Mt_ and EsxH_Mt_ arrest phagosome maturation

**Figure 4:**
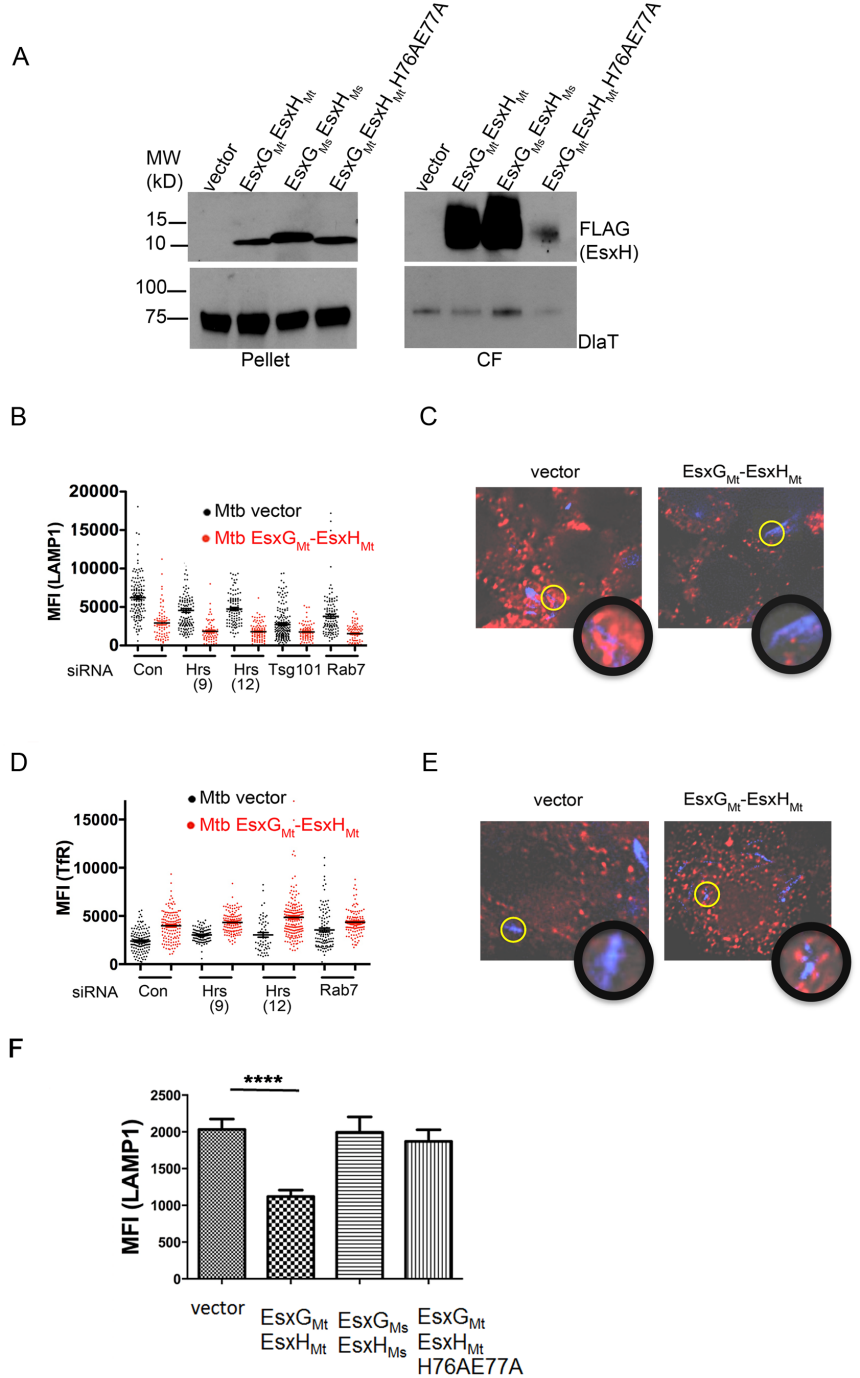
EsxG_Mt_ EsxH_Mt_ arrests phagosome maturation. (A) H37Rv transformed with empty vector, EsxG_Mt_ EsxH_Mt_-FLAG, EsxG_Ms_ EsxH_Ms-_FLAG or EsxG_Mt_ EsxH_Mt-_ H76A-E77A-FLAG were analyzed for the presence of EsxH in the pellet and culture filtrate (CF). DlaT (Rv2215), a cytosolic protein, was used as a loading control and to indicate the degree of bacterial lysis. (B) MFI of phagosomal LAMP1 24 hpi in RAW cells treated with siRNAs and infected with Mtb containing EsxG_Mt_-EsxH_Mt_ plasmid (red) or vector control (black); data points are the mean fluorescence intensity (MFI) around at least 70 phagosomes for each condition; *p*<0.0001 between the two Mtb strains for all conditions, unpaired Student's *t*-test. (C) Composite images of cells infected with Mtb with autofluorescence of Mtb (blue) and LAMP1 (red). Regions indicated by yellow circles are shown in higher magnification in adjacent panels. (D) MFI of phagosomal TfR 24 hpi in RAW cells treated with siRNAs and infected with Mtb containing EsxG_Mt_-EsxH_Mt_ plasmid (red) or vector control (black); data points are the mean fluorescence intensity (MFI) around at least 50 phagosomes for each condition; *p*<0.0001 between the two Mtb strains for all conditions, except Rab7 (p = 0.0005), unpaired Student's *t*-test. (E) Composite images of cells infected with Mtb with autofluorescence of Mtb (blue) and TfR (red). Regions indicated by yellow circles are shown in higher magnification. (F) MFI of phagosomal LAMP1 in RAW cells infected with Mtb containing the indicated plasmids 24 hpi. Bars show mean +/− SEM. ****p<0.0001, unpaired Student's *t*-test.

Because Esx-3 is essential for Mtb growth, we examined the effect of overexpressing EsxH_Mt_ on bacterial trafficking. First, we wanted to determine whether EsxG_Mt_ EsxH_Mt_ could confer a block in maturation to phagosomes containing Msmeg. However, when we expressed EsxG_Mt_ EsxH_Mt-_-FLAG under control of the hsp60 promoter in Msmeg, it was not secreted (Figure S6). It was secreted by Mtb (Figure 4A), and when we examined whether overexpression of EsxG_Mt_ and EsxH_Mt_-FLAG could enhance phagosome maturation arrest of Mtb, we found less co-localization between Mtb and LAMP1 and enhanced co-localization with TfR with the strain overexpressing EsxH_Mt_-FLAG, compared to a strain transformed with vector control (Figure 4B–E). The defect in lysosomal trafficking was similar to siRNA-mediated silencing of Hrs, Tsg101, and Rab7, and the combination of EsxG_Mt_ EsxH_Mt_ overexpression and ESCRT-silencing resulted in lower LAMP1 co-localization than either manipulation alone (Figure 4B). An Mtb strain that expressed EsxG_Mt_ EsxH_Mt-_ H76A-E77A did not exhibit altered trafficking, but the mutant protein also failed to be secreted (Figure 4A, 4F). Mtb did secrete EsxG_Ms_ EsxH_Ms_-FLAG, which, unlike EsxG_Mt_ EsxH_Mt_-FLAG, did not block LAMP1 co-localization (Figure 4A and 4F). We conclude that EsxG_Mt_ EsxH_Mt_, but not EsxG_Ms_ EsxH_Ms_, can prevent lysosomal trafficking during infection, most likely reflecting the ability of EsxH_Mt_ to bind Hrs and impair ESCRT activity.

## Discussion

We used high throughput Y2H interactome mapping to identify interactions between secreted Mtb proteins and human proteins, identifying 99 new potential interactions. We made use of a large body of literature that has attempted to catalogue the secretome of Mtb. Our study is subject to the uncertainty around the definition of the Mtb secretome. For example, proteins can be in the culture filtrate due to bacterial lysis, rather than secretion, and bioinformatics predictions may be inaccurate. In addition, many secreted proteins play an intrinsic role in the bacterial lifecycle and are unlikely to make a biologically meaningful interaction with host proteins. Thus, to estimate a false positive hit rate of our system, we included a non-secreted control collection. We found approximately two-fold enrichment in the rate of interactions comparing the secretome collection to the control collection, suggesting that true interactions were identified, but that there also may be a relatively high rate of “pseudo-interactions,” which may be valid biophysically but never occur *in vivo* because the involved proteins are separated spatially or temporally. In addition, the interactome list is by no means complete. We did not screen the entire putative secretome, but rather imposed criteria to try to arrive at a set that was enriched for true secreted proteins likely to play a role in virulence. In addition, the screen was not performed to saturation, and only a fraction of verifiable interactions can be detected by a single method to detect PPIs [31]. Therefore, the list is not comprehensive and likely contains false-positives, but given the paucity of data on host-pathogen interactions in Mtb, it has likely significantly expanded the known Mtb-human protein-protein interactome. It represents a resource for investigators working on Mtb; the confirmation and significance of such interactions will require further validation.

Interactome mapping provides an unbiased strategy to identify host-pathogen interactions for pathogens in which genetic strategies are limited, and it can be complemented by depletion studies in host cells. Such physical interaction mapping can identify redundant or essential factors that may be missed using genetic approaches. For example, in the case of EsxH_Mt_, its importance may have been unrecognized in previous genetic approaches to identify Mtb virulence factors because of redundancy within this large gene family, the existence of additional mechanisms to modulate phagosome maturation, and the essentiality of the Esx-3 system.

This Y2H screen and our previous genome-wide RNAi screen in *Drosophila* pointed to the importance of the ESCRT machinery in mycobacterial pathogenesis. Here, we show that the ESCRT machinery is important in restricting the intracellular growth of pathogenic mycobacteria, which likely reflects a role of ESCRT in trafficking bacteria to the lysosome, although the effects of ESCRT on endo-lysosomal content and signaling pathways may also play a role. In addition, by modulating the ESCRT machinery, Mtb might alter antigen presentation or exosome formation [42], [47].

Further work is required to understand exactly how EsxH_Mt_ impairs ESCRT function. We envision that EsxH_Mt_ inhibits ESCRT on or near the mycobacterial phagosome, where its local concentration would be highest, as opposed to globally disrupting ESCRT. The C-terminal half of Hrs, which we showed binds EsxH_Mt_, has previously been shown to be involved in the interactions with Tsg101 [48], [49], STAM [50], and SNAP-25 [45]. Thus, one possibility is that EsxH_Mt_ interferes with these associations. The relatively low affinity measured *in vitro* between EsxH_Mt_ and Hrs (∼5 µM) may be sufficient to disrupt Hrs interactions with other host proteins, as the interactions of Hrs with many of its binding partners are of low affinity [45], [48], [51], [52]. For example, HIV Gag recruits Tsg101 to sites of viral budding by binding the Tsg101 UEV domain with an even lower affinity (K_D_∼21–50 µM) [48], [53]. There may also be a particular form of Hrs or EsxH_Mt_ that exist *in vivo* in macrophages that exhibits higher affinity. For example, Hrs interacts with the endosomal membrane, engages in numerous protein-protein interactions, and is modified by phosphorylation and ubiquitination, none of which occur when the affinity is measured with recombinant protein. Interestingly MG132, which is known to alter ESCRT activity [54], [55], enhanced our ability to detect an interaction between EsxG_Mt_-EsxH_Mt_ and Hrs in co-immunoprecipitation experiments in HEK293 cells. One explanation for the requirement of MG132 to detect the Hrs-EsxH_Mt_ interaction by co-immunoprecipitation may be related to the observation that MG132 impairs ESCRT-dependent trafficking [54], [55]. Thus, it is possible that MG132 stabilizes the interaction between EsxH_Mt_ and Hrs by altering ESCRT, although other potential mechanisms could be envisioned. Even in the absence of MG132, EsxG_Mt_-EsxH_Mt_ inhibits ESCRT function. Therefore, we speculate that EsxH_Mt_ preferentially binds to a form of Hrs that exists transiently in cells, a form that is stabilized by MG132. Once bound to Hrs, EsxH_Mt_ could interact with other host proteins that modify Hrs or ESCRT components.

Hrs is one of several host factors that Mtb likely target to create a protected niche [3], [4]. Lipoamide dehydrogenase (LpdC) is thought to prevent phagosome-lysosome maturation by retaining the host factor, coronin 1 [29], [56]. PtpA, a secreted tyrosine phosphatase, may directly exclude the vacuolar-H^+^ATPase during infection, impairing acidification and phagosome maturation [27], [28], [57], while nucleoside diphosphate kinase A (NdkA) targets Rab7 activation [30], [58]. In addition, there is less phosphatidylinositol 3-phosphate on the mycobacterial phagosome than latex bead phagosomes, which may reflect the activity of the secreted lipid phosphatase, SapM [59], [60]. This leads to impaired recruitment of Hrs [39]. Thus, Hrs activity could be inhibited on mycobacterial phagosomes by two synergistic mechanisms: impaired recruitment and direct targeting by EsxH_Mt_. How the activities of these various bacterial effectors are coordinated, whether they are required in concert or function in different cell types or at different time points post-infection, has not been explored. In order to evaluate the relative contribution of the EsxH_Mt_-Hrs interaction to trafficking and intracellular survival during infection, we will have to identify mutations in EsxH_Mt_ that disrupt its binding to Hrs, but that do not interfere with its secretion from Mtb or disrupt bacterial iron acquisition.

It was surprising to us that it was possible to alter Mtb trafficking by overexpressing EsxG_Mt_ EsxH_Mt_, as if Mtb normally expresses a “sub-optimal” amount to maximally alter phagosome maturation. Similarly, ESCRT and Rab7 appear to be sub-maximally inhibited, as further impairing their function by RNAi-mediated silencing also enhances the block in phagosome-lysosome fusion. Given that over-expression of EsxH_Mt_ by Mtb caused a greater effect on trafficking than Hrs silencing (Fig. 4B), EsxH_Mt_ may have additional cellular targets involved in cellular trafficking as well. One explanation for the observation that additional EsxH_Mt_ can further impair trafficking is that this reflects *in vitro* growth conditions, whereas, during infection *in vivo*, EsxH_Mt_ levels may be higher. An additional possibility is that EsxG_Mt_ EsxH_Mt_ production is finely tuned to balance an opposing effect that is detrimental to the bacteria. For example, EsxG_Mt_ and EsxH_Mt_ generate prominent T cell responses, [14], [15]. In addition, we found that although the Mtb strain that overexpresses EsxG_Mt_ EsxH_Mt_ exhibited diminished co-localization with LAMP1 and enhanced co-localization with TfR, there was no difference in intracellular growth for this strain relative to control (Figure S7). Thus, overexpression of EsxG_Mt_ EsxH_Mt_, while promoting trafficking, might come with an opposing intracellular fitness cost for bacteria.

In summary, our studies demonstrate that Mtb adapted Esx-3, an ancient microbial system for iron acquisition, to alter host cell physiology. Analogously, Esx-1, which is important for conjugation in Msmeg [61], mediates important host interactions that are critical for virulence, including permeabilizing the mycobacterial phagosome and altering phagosome maturation [5]–[9], [62]. Thus, the duplication and adaptation of TSSSs to new functions appears to be a particularly important evolutionary path to virulence in Mtb. The relatively low affinity between Hrs and its endogenous binding partners may have made it particularly susceptible to manipulation by diverse pathogens, from enveloped viruses to Mtb. The observation that macrophage control of infection is especially sensitive to ESCRT inhibition suggests ESCRT is a likely target of additional pathogens as well.

## Materials and Methods

Detailed methods, including description of Y2H interaction mapping, plasmids, siRNAs, and Hill plot analysis, are provided in Text S1.

### Tissue culture conditions

RAW264.7 and HEK293 cells were grown in Dulbecco's Modified Eagle Medium (DMEM; Gibco), 20 mM HEPES, 2 mM L-glutamine, and 10% heat inactivated fetal bovine serum (hiFBS; Invitrogen). BMDMs were isolated from C57BL/6 mice as described [63] Penicillin/Streptomycin (Gibco), added for passaging, was omitted during infections. A549 cells were grown in RPMI 1640 Medium (Gibco), 2 mM L-glutamine, 1× Non-essential Amino Acids (Cellgro), and 10% hiFBS. Cells were grown at 37°C with 5% CO_2_ atmosphere. siRNAs were transfected with Hiperfect (Qiagen). Plasmids were transfected into HEK293 cells with Effectene (Qiagen) and A549 cells with Lipofectamine 2000 (Invitrogen).

### Bacterial strains and growth conditions

*M. tuberculosis* H37Rv, *M. bovis-BCG*, and *M. smegmatis* mc^2^155 were grown at 37°C to log phase in Middlebrook 7H9 media with 0.05% Tween 80, BBL Middlebrook OADC Enrichment, and 0.2% glycerol. Plasmids were selected with 50 µg/ml kanamycin or hygromycin depending upon the resistance marker. To generate EsxG_Mt_ EsxH_Mt_-FLAG and EsxG_Ms_ EsxH_Ms_-FLAG for overexpression in mycobacteria, EsxG-EsxH was amplified from BCG (the EsxG-EsxH region is 100% identical between BCG and Mtb) and Msmeg genomic DNA, respectively, using primers described in Text S1. The PCR products were cloned into pSYMP under control of the *hsp60* promoter [64].

### Intracellular bacterial growth assay

RAW cells were seeded one day before infection or they were transfected with siRNAs two days prior to infection with a single cell suspension of Mtb (MOI∼2–5), obtained as previously described [40]. The cells were extensively washed and lysed with 0.2% Triton X-100 3 hpi or 2d later and serial dilutions were plated on 7H10 or 7H11. CFU were calculated 15 to 21 d later.

### Lysosomal trafficking assay

RAW cells were transfected with siRNAs for two days and then infected with a single cell suspension of BCG or Mtb (MOI∼20) for 3 h, then washed extensively. Cells were fixed 24 hpi with 4% formaldehyde/PBS for BCG and with 1% paraformaldehyde/PBS overnight for Mtb and immunostained for LAMP1 (Abcam) or TfR (Invitrogen). For Lysotracker (Invitrogen) staining, unfixed RAW cells were incubated with 200 nM Lysotracker, washed twice in PBS, and visualized. Images were captured using the Nikon Eclipse TiE/B automated fluorescent microscope with Photometrics HQ@ Monochrome digital camera. 60× z-stack images were acquired, deconvoluted, and analyzed using NIS-Elements DUO software (see Fig. S3 for details). Contrast was not altered prior to automated image analysis; for reproduced images, alterations were applied equally to all samples.

### Recombinant protein binding assay

His-tagged EsxG_Mt_-EsxH_Mt_ was purified as described in Text S1. Prior to inclusion of recombinant proteins in binding reactions they were centrifuged at 100,000× g for 30 min to remove aggregated protein. To determine whether EsxG_Mt_ EsxH_Mt_ binds to Hrs in a direct and saturable manner, 1.0 µg EsxG_Mt_ EsxH_Mt-_-6XHis was bound to Ni-NTA beads and incubated with increasing amounts of purified, soluble Hrs (0∼4 µg) in 20 mM HEPES [pH, 7.4], 150 mM KCl, and 0.05% Tween-20, with protease inhibitors (10 mM leupeptin, 1 µg/µL pepstatin, 0.3 mM aprotinin, and 1.74 µg/µL PMSF) for 1 h at 4°C. Beads were washed in PBST (0.1 M PBS 0.05% Tween-20) with 10 mM imidazole. Bound Hrs was analyzed by SDS-PAGE and Coomassie staining. Bands were subject to quantification with ImageJ software (v. 1.42).

### Co-immunoprecipitation and Western blotting

Cellular lysates were prepared in RIPA buffer with Halt Protease Inhibitor Cocktail (Thermo Scientific) and 10 mM N-ethylmaleimide (Sigma) and analyzed by western blotting. The antibodies used for western analysis are: actin (clone C4/MAB1501, Millipore), Hrs (M79/sc-30221, Santa Cruz Biotechnology), Rab7 (117, Abcam), FLAG (F7425, Sigma), EGFR (#4267S, Cell Signaling), and FK2 (Millipore). For co-immunoprecipitation, HEK293 cells transfected with Hrs-myc and Esx expression plasmids were treated with 20 µM MG132 (Calbiochem) for 3 h prior to mechanical lysis and incubated with Dynabeads Protein G (Novex, Life technologies) pre-bound to isotype control antibody (sc-2025, Santa Cruz Biotechnology), anti-myc antibody (sc-40/9E10, Santa Cruz Biotechnology), or anti-V5 antibody (Invitrogen), and bound proteins were analyzed by western blotting.

### EGFR and EGF degradation assays

Two days after transfection, HEK293 cells were incubated in serum-free DMEM and treated with 100 ng/ml of recombinant human EGF (rh-EGF, R&D Systems) essentially as described [65]. Cells were harvested immediately prior to addition of EGF and 90 min later and EGFR analyzed by western blotting. EGF trafficking was assessed similarly to described [66]. Two days after transfection of A549 cells with siRNA or DNA, cells were incubated with serum-free RPMI before addition of 25 µg/ml Alexa Fluor 488-EGF (Invitrogen) in EGF uptake media (RPMI, 2% BSA, 20 mM HEPES) at 4°C for 1 h. Cells were washed to remove unbound ligand, incubated at 37°C for 90 min, and examined by immunofluorescence microscopy.

### Mycobacterial secretion of EsxH

To analyze secretion of EsxH_Ms_-FLAG or EsxH_Mt_-FLAG from Mtb and Msmeg, strains were grown to mid-log phase, washed with PBS, and inoculated into Sauton's media. In Sauton's media, they were grown to reach log phase (overnight in the case of Msmeg and for two days in case of Mtb). Thereafter, mycobacteria were pelleted by centrifugation. The supernatants were filtered through 0.22 µM filters followed by precipitation with 12% trichloroacetic acid. The precipitate was washed with ice-cold acetone, air dried, and resuspended in SDS sample buffer. The bacterial pellets were lysed by bead beating in lysis buffer (50 mM Tris-HCl pH 7.5, 5 mM EDTA, 0.6% SDS, 10 mM NaH_2_PO_4_, and protease inhibitor) with 0.1 mm zirconia/silica beads (BiosSpec Products, Inc.). SDS-sample buffer was added, followed by boiling at 95°C for 5 min. Antibody to the pyruvate dehydrogenase E2 component sucB (Rv2215/dlaT) [67], a cytosolic protein, was used as a loading control and to indicate the degree of bacterial lysis.

## Supporting Information

Dataset S1 **Secretome collection screened.**
(XLS)

Dataset S2 **Control collection screened.**
(XLS)

Dataset S3 **Y2H interactions between Mtb secretome and human ORFs.**
(XLS)

Figure S1 **siRNA-mediated depletion of Hrs and Rab7.** (A) RAW264.7 (RAW) cells were treated with 50 nM ON-TARGET*plus* individual siRNAs (#9–#12) targeting Hrs or control for 2 d. (B) RAW cells were treated with increasing concentration of siRNA#9 targeting Hrs for 2 or 5 days. (C) A549 cells treated with 50 nM Hrs siRNAs (#12) or control for 2 d. (A)–(C) Western blotting with antibody recognizing Hrs was used to assess silencing. (D) RAW cells were treated with 30 nM siRNA targeting Rab7 or control. Silencing was assessed 2 d later by western blotting using an antibody recognizing Rab7. (E) RAW cells treated with control siRNA (siCON) or siRNA targeting Hrs (#9 or #12) for 2 d were examined by immunofluorescence using antibodies against Hrs, shown in red, and ubiquitinated proteins (FK2) in green.
(TIF)

Figure S2 **siRNAs targeting Hrs and Rab7 enhance the intracellular survival of BCG in BMDMs.** 4×10^4^ BMDMs were transfected with 30 nM siRNA pools targeting Hrs (ON-TARGET*plus*) or Rab7 (siGENOME) 6–8 d after harvest. 3 d later, they were infected with BCG (MOI of 2 to 5). CFU were enumerated 2 days post-infection and are normalized to the average number of CFU in control wells from two independent experiments. Results reflect the mean +/− SEM. *p<0.05; **p<0.01, unpaired Student's *t*-test.
(TIF)

Figure S3 **Automated image analysis of phagosome maturation** (A) For quantifying the degree of co-localization between bacteria and cellular markers or Lysotracker, images were background subtracted and analyzed using the Binary Operation Analysis within NIS Elements Software. Bacteria were selected in the green channel. The region the software has selected that corresponds to the bacteria is shown in red in the second panel. That region was expanded (dilate binary) and then eroded and a binary operation was performed to generate a “donut” in the region surrounding the bacteria. The region of interest (ROI) is shown in purple. The mean fluorescence intensity (MFI) in the ROI was determined for the cellular marker. Bacteria were analyzed from at least three fields per sample per experiment. We confirmed that automated quantification closely paralleled manual quantification and visual scoring by a blinded observer. (B) To further validate the automated analysis, we verified enhanced LAMP1 co-localization in macrophages pre-treated with IFN-γ, which promotes phagosome maturation [61]. RAW cells treated with control siRNA (siCON) were either pre-treated with IFN-γ or solvent control 24 hours prior to infection with Mtb-GFG. In IFN-γ pre-treated macrophages there is a significant shift in LAMP1 co-localization around bacterial phagosomes 24 hpi. Data points are the MFI of LAMP1 around bacteria; bars show mean +/− SEM; *p*<0.0001. (C) Co-localization of Lamp1, Lysotracker, and TfR with metabolically active BCG compared to co-localization with total BCG. RAW cells were treated with control siRNA (siCON) and infected with BCG constitutively expressing GFP (BCG-GFP) or BCG expressing GFP under a tetracycline inducible promoter (BCG-tet-GFP). AnTc was added 24 hpi to induce expression of GFP. Because it takes >12 h for the strain to become detectably GFP positive, co-localization between BCG-tet-GFP and LAMP1, LysoTracker, or TfR was measured at 48 hpi. For the BCG-GFP strain, LAMP1 and TfR were examined at 48 hpi and LysoTracker at 24 hpi. Data points are the MFI around bacteria; bars show mean +/− SEM; *p* value of BCG-tet-GFP compared to BCG-GFP for LAMP1 = 0.0081, for LysoTracker <0.0001, for TfR = 0.0046.
(TIF)

Figure S4 **Quantification of EsxH_Mt_–FLAG in transfected HEK293 cells.** EsxH_Mt_ was co-transfected with vector control, EsxG_Mt_, or Hrs as indicated. Prior to protein harvest, cells were treated with DMSO or MG132. EsxH_Mt_-FLAG levels were quantified from at least three independent experiments using ImageJ software. *p<0.05; **p<0.01, unpaired Student's *t*-test; ns- not significant. Whiskers reflect the minimum and maximum data points, while the cross bars show the median.
(TIF)

Figure S5 **Treatment with MG132 does not result in higher molecular weight forms of the EsxH proteins.** HEK293 cells were transfected with plasmids as indicated. Cells were either treated with DMSO or MG132 prior to protein harvest. Lysates were examined for mono- and polyubiquitinated proteins using the FK2 antibody. The EsxH proteins were visualized using the FLAG antibody. No differences were seen in the mobility of EsxH_Mt_, EsxH_Ms_, or EsxH_Mt_-H76AE77A in the presence of MG132.
(TIF)

Figure S6 **EsxG_Mt_ EsxH_Mt_-FLAG is not secreted by Msmeg.** Msmeg transformed with empty vector, EsxG_Mt_ EsxH_Mt_-FLAG, or EsxG_Ms_ EsxH_Ms_-FLAG were analyzed for the presence of EsxH in the pellet and culture filtrate (CF). DlaT (Rv2215), a cytosolic protein, was used as a loading control and to indicate the degree of bacterial lysis.
(TIF)

Figure S7 **EsxG_Mt_ EsxH_Mt_-FLAG does not alter intracellular growth of Mtb.** RAW cells were infected with Mtb containing vector control, EsxG_Mt_ EsxH_Mt_-FLAG, or EsxG_Ms_ EsxH_Ms_-FLAG and bacterial CFU were enumerated at 3 h, 24 h and 48 h post-infection. No statistically significant differences were seen at any time point. Results reflect the mean +/− SEM.
(TIF)

Table S1 **Interactions identified between Mtb proteins.**
(DOCX)

Table S2 **Hit rate by category of Mtb ORFs.**
(DOCX)

Text S1 **Supporting text.** This file contains detailed methods, including description of Y2H interaction mapping, plasmids, siRNAs, and Hill plot analysis.
(DOCX)
