# Supplementary figures and images for: Mycobacterium tuberculosis Type VII Secreted Effector EsxH Targets Host ESCRT to Impair Trafficking
Source: PLoS Pathog. 2013 Oct 31;9(10):e1003734. doi: 10.1371/journal.ppat.1003734 (PMC3814348; doi:10.1371/journal.ppat.1003734)

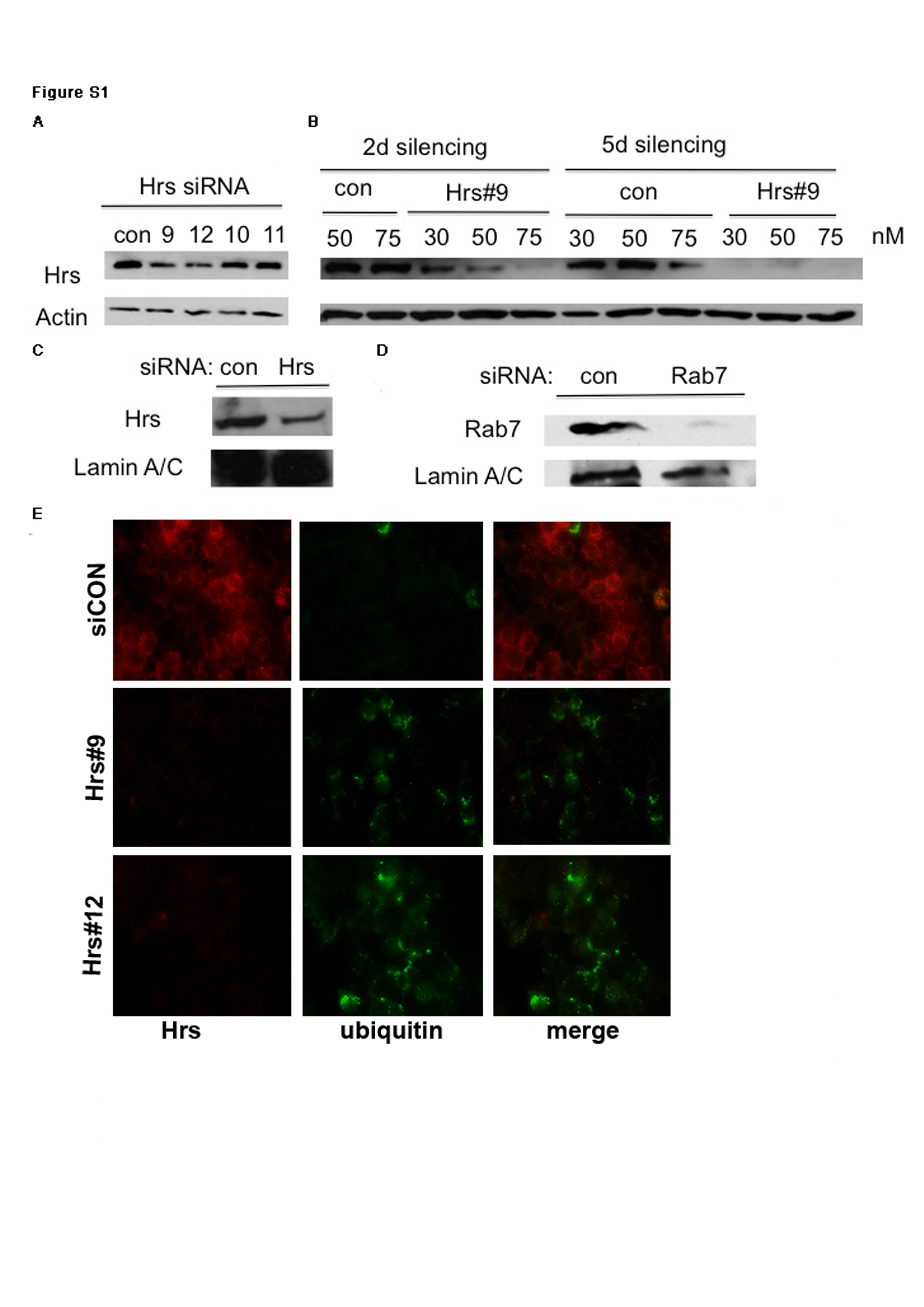

Supplement: Figure S1 — siRNA-mediated depletion of Hrs and Rab7. (A) RAW264.7 (RAW) cells were treated with 50 nM ON-TARGETplus individual siRNAs (#9–#12) targeting Hrs or control for 2 d. (B) RAW cells were treated with increasing concentration of siRNA#9 targeting Hrs for 2 or 5 days. (C) A549 cells treated with 50 nM Hrs siRNAs (#12) or control for 2 d. (A)–(C) Western blotting with antibody recognizing Hrs was used to assess silencing. (D) RAW cells were treated with 30 nM siRNA targeting Rab7 or control. Silencing was assessed 2 d later by western blotting using an antibody recognizing Rab7. (E) RAW cells treated with control siRNA (siCON) or siRNA targeting Hrs (#9 or #12) for 2 d were examined by immunofluorescence using antibodies against Hrs, shown in red, and ubiquitinated proteins (FK2) in green. (TIF) [file ppat.1003734.s004.tif]

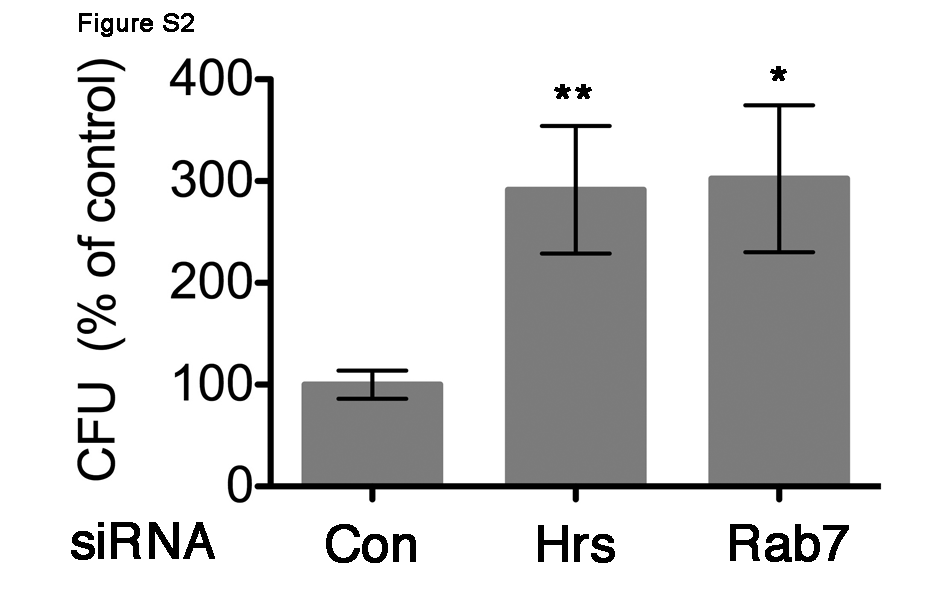

Supplement: Figure S2 — siRNAs targeting Hrs and Rab7 enhance the intracellular survival of BCG in BMDMs. 4×10^4^ BMDMs were transfected with 30 nM siRNA pools targeting Hrs (ON-TARGETplus) or Rab7 (siGENOME) 6–8 d after harvest. 3 d later, they were infected with BCG (MOI of 2 to 5). CFU were enumerated 2 days post-infection and are normalized to the average number of CFU in control wells from two independent experiments. Results reflect the mean +/− SEM. *p<0.05; **p<0.01, unpaired Student's t-test. (TIF) [file ppat.1003734.s005.tif]

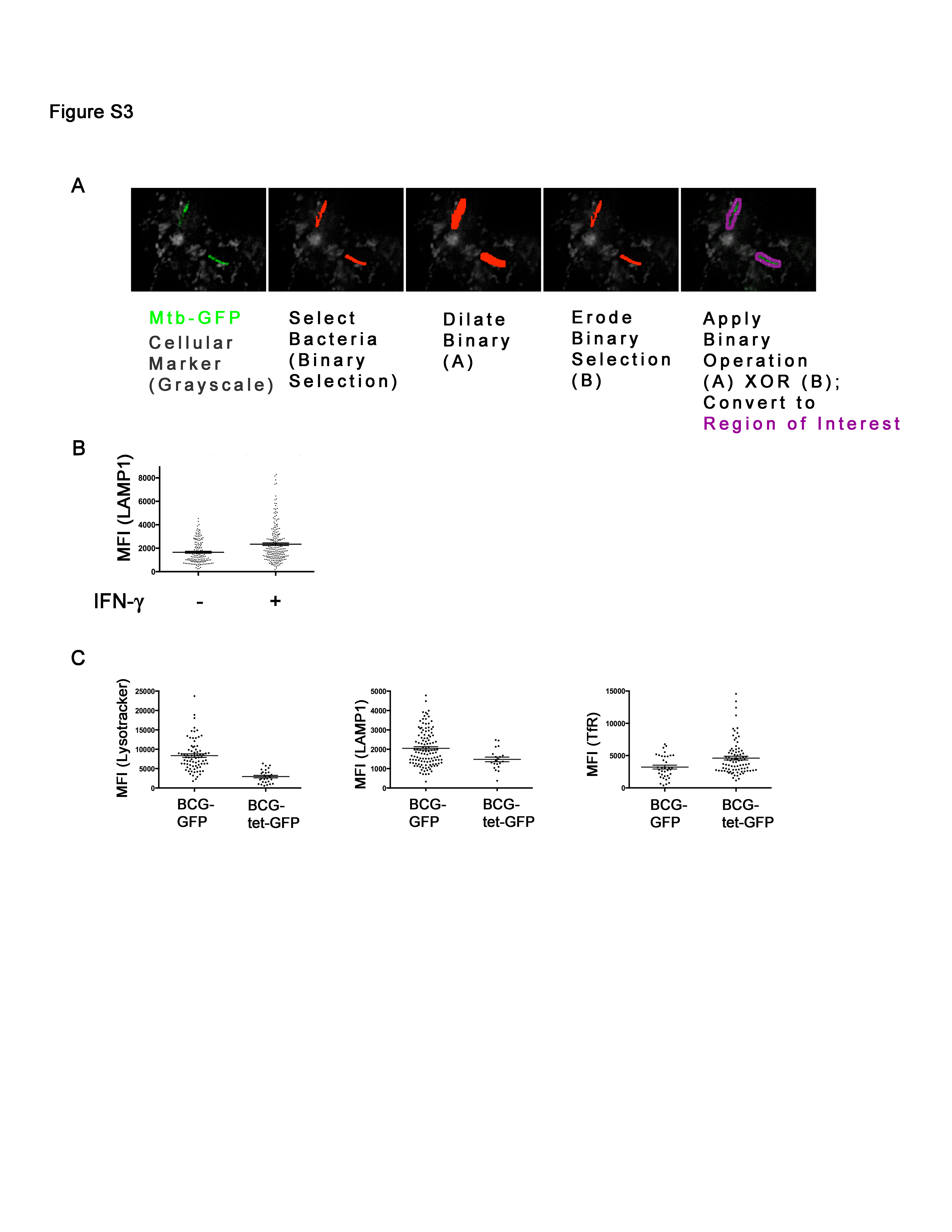

Supplement: Figure S3 — Automated image analysis of phagosome maturation (A) For quantifying the degree of co-localization between bacteria and cellular markers or Lysotracker, images were background subtracted and analyzed using the Binary Operation Analysis within NIS Elements Software. Bacteria were selected in the green channel. The region the software has selected that corresponds to the bacteria is shown in red in the second panel. That region was expanded (dilate binary) and then eroded and a binary operation was performed to generate a “donut” in the region surrounding the bacteria. The region of interest (ROI) is shown in purple. The mean fluorescence intensity (MFI) in the ROI was determined for the cellular marker. Bacteria were analyzed from at least three fields per sample per experiment. We confirmed that automated quantification closely paralleled manual quantification and visual scoring by a blinded observer. (B) To further validate the automated analysis, we verified enhanced LAMP1 co-localization in macrophages pre-treated with IFN-γ, which promotes phagosome maturation [61]. RAW cells treated with control siRNA (siCON) were either pre-treated with IFN-γ or solvent control 24 hours prior to infection with Mtb-GFG. In IFN-γ pre-treated macrophages there is a significant shift in LAMP1 co-localization around bacterial phagosomes 24 hpi. Data points are the MFI of LAMP1 around bacteria; bars show mean +/− SEM; p<0.0001. (C) Co-localization of Lamp1, Lysotracker, and TfR with metabolically active BCG compared to co-localization with total BCG. RAW cells were treated with control siRNA (siCON) and infected with BCG constitutively expressing GFP (BCG-GFP) or BCG expressing GFP under a tetracycline inducible promoter (BCG-tet-GFP). AnTc was added 24 hpi to induce expression of GFP. Because it takes >12 h for the strain to become detectably GFP positive, co-localization between BCG-tet-GFP and LAMP1, LysoTracker, or TfR was measured at 48 hpi. For the BCG-GFP strain, LAMP1 and Tf [file ppat.1003734.s006.tif]

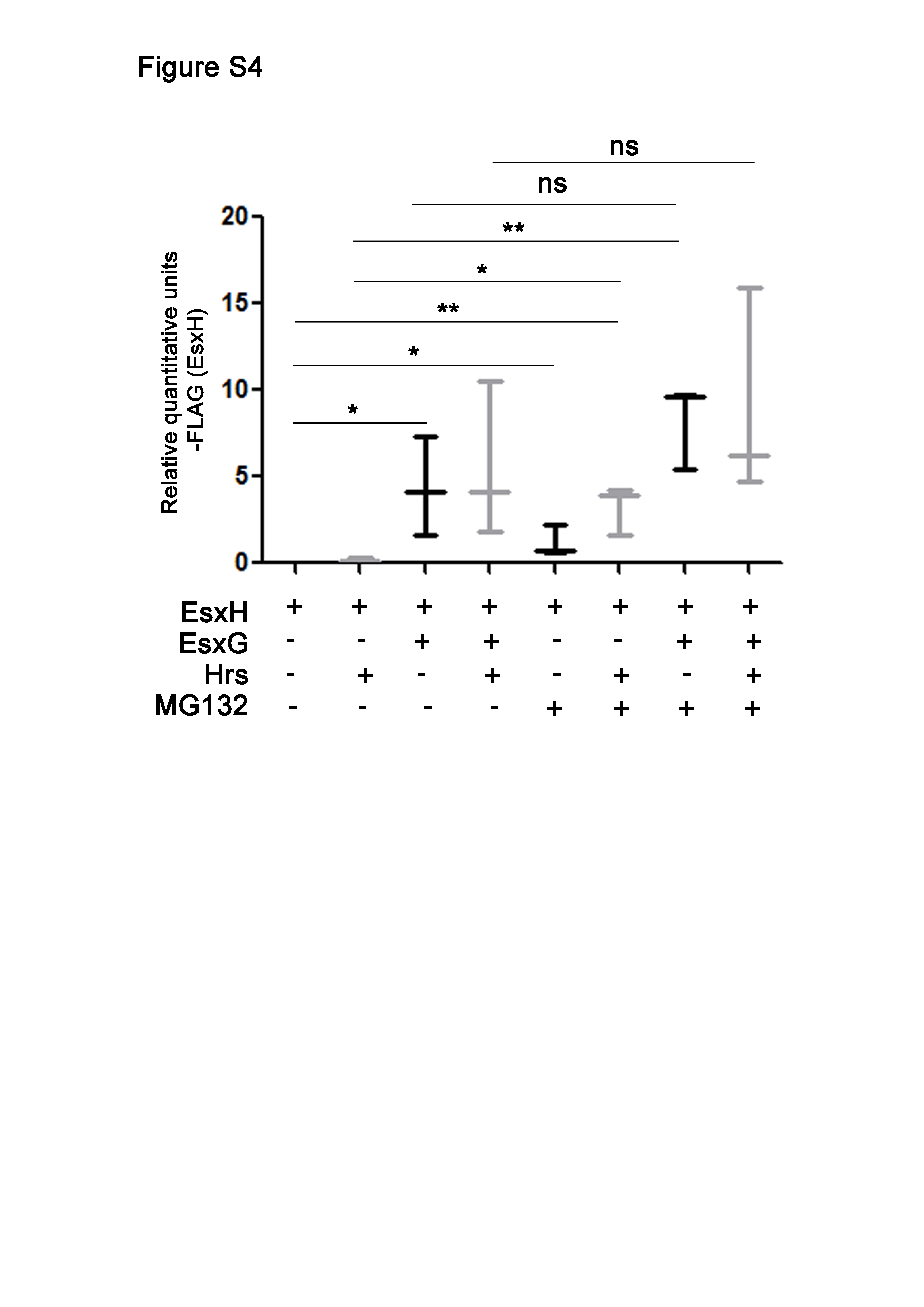

Supplement: Figure S4 — Quantification of EsxHMt–FLAG in transfected HEK293 cells. EsxHMt was co-transfected with vector control, EsxGMt, or Hrs as indicated. Prior to protein harvest, cells were treated with DMSO or MG132. EsxHMt-FLAG levels were quantified from at least three independent experiments using ImageJ software. *p<0.05; **p<0.01, unpaired Student's t-test; ns- not significant. Whiskers reflect the minimum and maximum data points, while the cross bars show the median. (TIF) [file ppat.1003734.s007.tif]

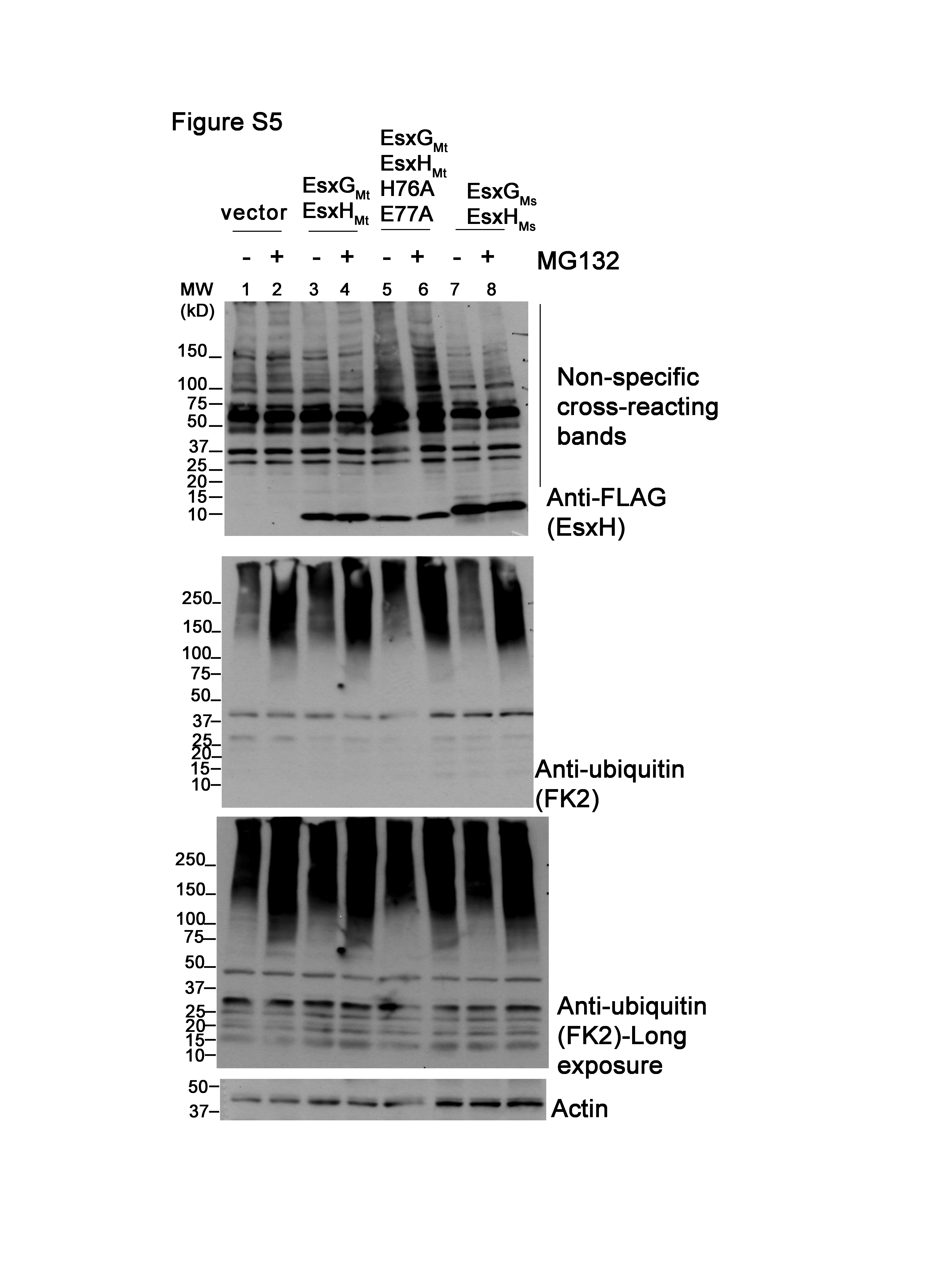

Supplement: Figure S5 — Treatment with MG132 does not result in higher molecular weight forms of the EsxH proteins. HEK293 cells were transfected with plasmids as indicated. Cells were either treated with DMSO or MG132 prior to protein harvest. Lysates were examined for mono- and polyubiquitinated proteins using the FK2 antibody. The EsxH proteins were visualized using the FLAG antibody. No differences were seen in the mobility of EsxHMt, EsxHMs, or EsxHMt-H76AE77A in the presence of MG132. (TIF) [file ppat.1003734.s008.tif]

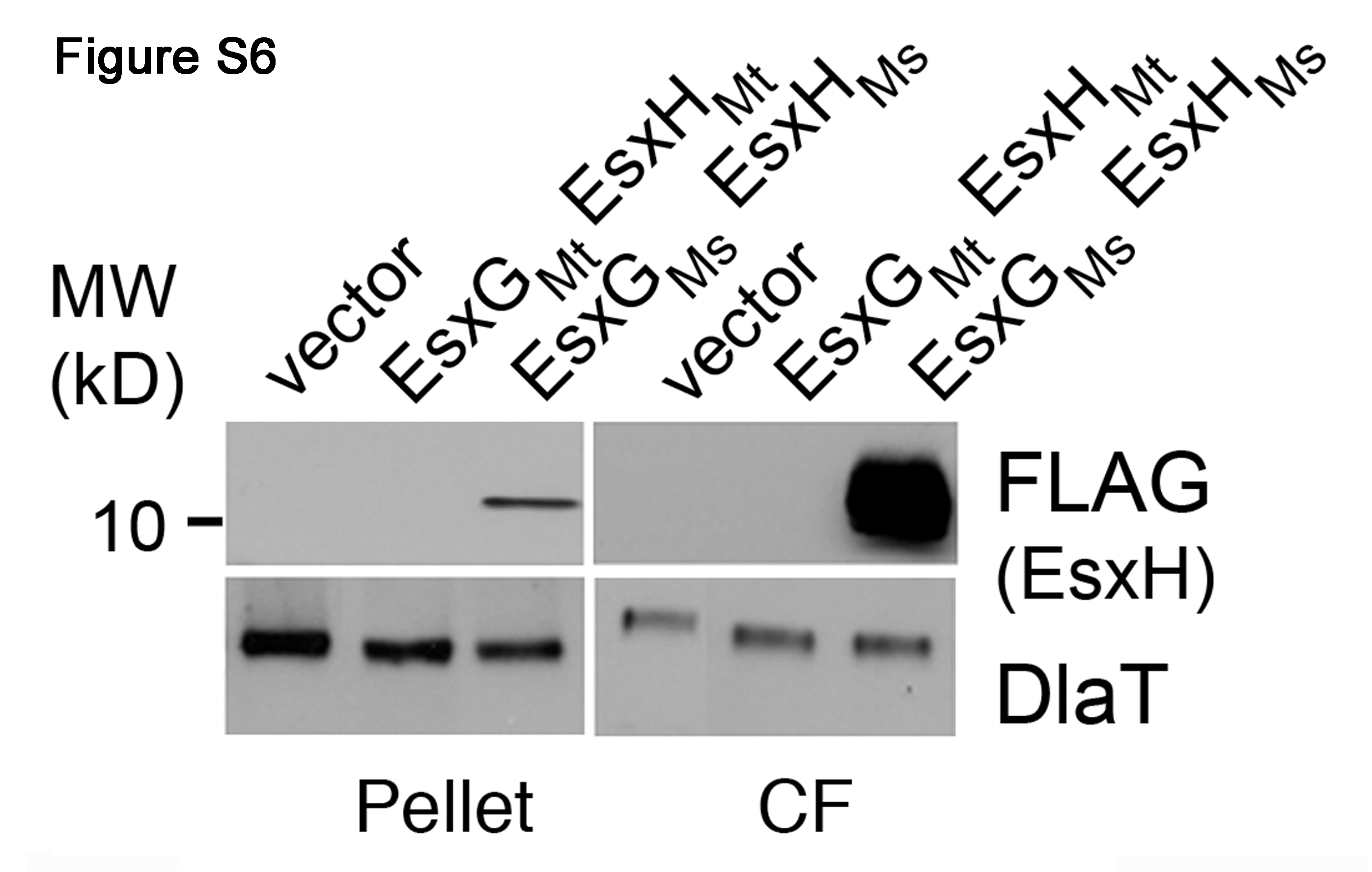

Supplement: Figure S6 — EsxGMt EsxHMt-FLAG is not secreted by Msmeg. Msmeg transformed with empty vector, EsxGMt EsxHMt-FLAG, or EsxGMs EsxHMs-FLAG were analyzed for the presence of EsxH in the pellet and culture filtrate (CF). DlaT (Rv2215), a cytosolic protein, was used as a loading control and to indicate the degree of bacterial lysis. (TIF) [file ppat.1003734.s009.tif]

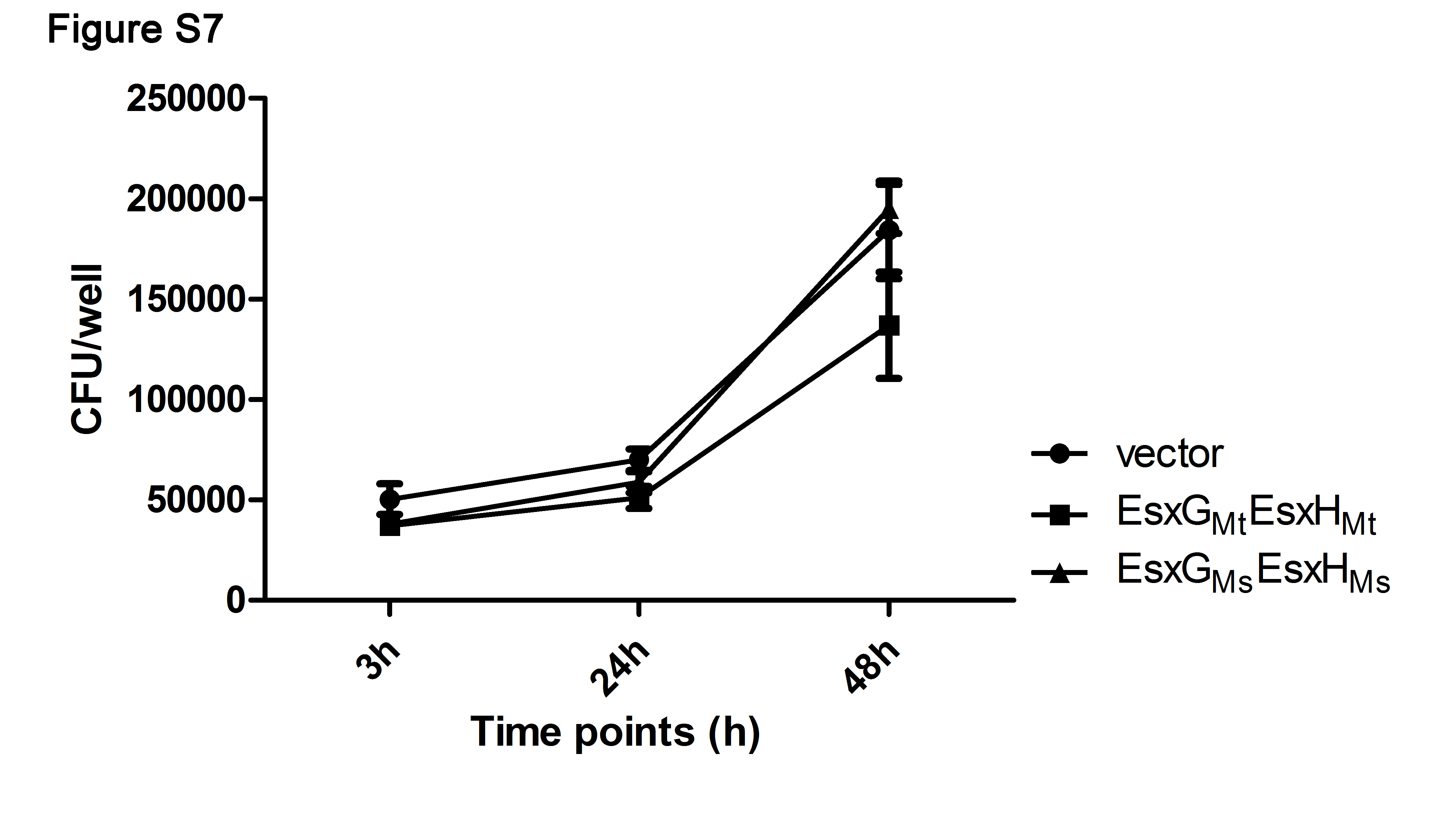

Supplement: Figure S7 — EsxGMt EsxHMt-FLAG does not alter intracellular growth of Mtb. RAW cells were infected with Mtb containing vector control, EsxGMt EsxHMt-FLAG, or EsxGMs EsxHMs-FLAG and bacterial CFU were enumerated at 3 h, 24 h and 48 h post-infection. No statistically significant differences were seen at any time point. Results reflect the mean +/− SEM. (TIF) [file ppat.1003734.s010.tif]
