# Supplementary material for: Mycobacterium tuberculosis Type VII Secreted Effector EsxH Targets Host ESCRT to Impair Trafficking
Source: PLoS Pathog. 2013 Oct 31;9(10):e1003734. doi: 10.1371/journal.ppat.1003734 (PMC3814348; doi:10.1371/journal.ppat.1003734)
Supplement: Table S1 — Interactions identified between Mtb proteins. (DOCX) [file ppat.1003734.s011.docx]

| Mtb ORF (DB) | Mtb ORF (AD) | Number of times identified |
| --- | --- | --- |
| Rv0831 (s)# | Rv0831 (s) | 1 |
| BfrB (s) | BfrB (s) | 2 |
| SseA (ns) | SseA (ns) | 1 |
| PhoY2 (s) | PhoY2 (s) | 1 |
| Rv3406 (ns) | Rv3406 (ns) | 1 |
| Rv1531 (s) | Rv1531 (s) | 1 |
| Rv1636 (s) | Rv3531c (ns) | 1 |
| EsxS (s) | EsxH (s) | 1 |
| EsxR (s) | EsxG (s) | 2 |
| EsxW (s) | EsxO (s) | 5 |
| EsxP (s) | EsxO (s) | 3 |
| EsxW (s) | EsxN (s) | 1 |
| EsxA (s) | EsxB (s) | 1 |
| EsxW (s) | EsxL (s) | 1 |

**Table S1. Interactions Identified Between Mtb Proteins.**

# letter in parenthesis indicates whether protein is predicted to be secreted (s) or not (ns). The table shows those Mtb ORFs that interacted in the Y2H when expressed as DB and AD fusions. The number of times an interaction was identified is indicated.
