# Supplementary material for: Mycobacterium tuberculosis Type VII Secreted Effector EsxH Targets Host ESCRT to Impair Trafficking
Source: PLoS Pathog. 2013 Oct 31;9(10):e1003734. doi: 10.1371/journal.ppat.1003734 (PMC3814348; doi:10.1371/journal.ppat.1003734)
Supplement: Table S2 — Hit rate by category of Mtb ORFs. (DOCX) [file ppat.1003734.s012.docx]

| Mtb ORF category | Number in collection | Number with  PPIs with human ORFs | Hit rate | p-value* |
| --- | --- | --- | --- | --- |
| Secretome collection | 339 | 53 | 0.16 | ns |
| Control collection | 60 | 5 | 0.08 | N/A |
| Found in CF | 285 | 45 | 0.16 | ns |
| Predicted signal sequence | 182 | 29 | 0.16 | ns |
| Found in CF and predicted signal sequence | 139 | 23 | 0.17 | ns |
| Secreted based upon phoA or b-lac fusion | 30 | 8 | 0.27 | 0.028 |
| Predicted Tat-dependent secretion | 21 | 1 | 0.05 | ns |
| SecA2-dependent secretion | 3 | 1 | 0.33 | ns |
| Esx family member | 16 | 6 | 0.38 | 0.0087 |
| **Functional Category** |  | | | |
| Virulence, detoxification, adaptation (0) | 26 | 5 | 0.19 | ns |
| Lipid metabolism (1) | 9 | 2 | 0.22 | ns |
| Information pathways (2) | 1 | 0 | 0 | ns |
| Cell wall and cell processes (3) | 134 | 29 | 0.22 | 0.025 |
| Insertion sequences or phages (5) | 2 | 0 | 0 | ns |
| PE/PPE (6) | 11 | 0 | 0 | ns |
| Intermediary metabolism and respiration (7) | 31 | 2 | 0.06 | ns |
| Regulatory proteins (9) | 11 | 3 | 0.12 | ns |
| Conserved hypotheticals (10) | 94 | 11 | 0.12 | ns |
| Conserved hypotheticals with an orthologue in *M. bovis* (16) | 20 | 1 | 0.05 | ns |

**Table S2. Hit Rate by Category of Mtb ORFs**

* Fisher’s exact test compared to control collection; ns- not significant. Mtb ORFs that interacted with human ORFs were categorized based upon listed attributes. The hit rate in each category was compared to the hit rate of the control collection.
