# Supplementary material for: Mycobacterium tuberculosis Type VII Secreted Effector EsxH Targets Host ESCRT to Impair Trafficking
Source: PLoS Pathog. 2013 Oct 31;9(10):e1003734. doi: 10.1371/journal.ppat.1003734 (PMC3814348; doi:10.1371/journal.ppat.1003734)
Supplement: Text S1 — Supporting text. This file contains detailed methods, including description of Y2H interaction mapping, plasmids, siRNAs, and Hill plot analysis. (DOCX) [file ppat.1003734.s013.docx]

**Text S1**

**Y2H Interactome Mapping**

The literature was curated to generate a list of all predicted secreted proteins from *Mycobacterium tuberculosis*. This included proteins found in culture filtrate from a variety of published studies [1-12] and the Proteome Database System for Microbial Research at Max Planck Institute for Infection Biology (<http://www.mpiib-berlin.mpg.de/2D-PAGE/>). It also included proteins predicted to have a signal sequence but lacking predicted transmembrane domains (those in the Top208-TM category) [13]. Proteins predicted to be secreted by a TAT-dependent mechanism from published studies [14,15] and TATFIND (<http://www.sas.upenn.edu/~pohlschr/tatprok.html>) were included, as were secA2-dependent proteins [16,17]. In addition, proteins found to be secreted based upon assayable protein fusions [18,19] and ESAT-6/CFP10-like proteins were part of the collection. In detailed study, a number of proteins have been shown or hypothesized to interact with host factor(s), and these were included [20-25]. Additional studies were reviewed to arrive at the final list [26-38]. Lastly, Tuberculist (<http://genolist.pasteur.fr/TubercuList/>) was searched using the word “secreted” and 116 hits were manually curated.

In order to generate a first generation collection for screening, the full list was annotated to remove certain ORFs. ORFs were removed if they had multiple TM domains based upon published annotations [2,39] or the TMHMM prediction tool in Tuberculist. They were also eliminated if they were found to be essential or cause slow growth [40], with the exceptions of PknG and LpdC. EsxG and EsxH were not identified as essential in Sassetti et al., 2003 and were therefore included in our collection. However, further study suggests that they are likely to be essential *in vitro* [41,42]. They were probably not identified in the Sassetti study for technical reasons because of the high degree of similarity between Esx proteins. The list was further curated to remove ORFs in functional category 1 (lipid metabolism), 2 (information pathway), and 7 (intermediary metabolism and respiration) unless they had possible signal sequences, possible TAT-dependent secretion, their expression was induced early in macrophages [43], they were required *in vivo* [44], or their corresponding mutants were attenuated in macrophages [45-47]. Although this may have removed true secreted genes, it enriched the collection for proteins found in CF by multiple studies; 75% of the eliminated ORFs encode proteins that were identified in CF in only a single proteomic study, in comparison to those that remained for which 32% were detected in only a single study. From the final list, 339 ORFs that were sequence validated were provided by PFGRC (Dataset S1). There were 35 additional ORFs provided by PFGRC that contained mutations that were also screened; none of these exhibited interactions with human proteins and they were excluded from subsequent analysis.

We anticipated that only a small fraction of the secretome collection would interact with human proteins, since many secreted proteins play an intrinsic role in the bacterial lifecycle. Thus, we included sixty ORFs that are not likely to be secreted to serve as controls to estimate a false positive hit rate of our system (Dataset S2). Most of the control collection were ORFs available at PFGRC that were chosen randomly from proteins found in the cellular protein (CP) fraction on <http://www.mpiib-berlin.mpg.de/2D-PAGE/> or found exclusively in the CP/cytosolic fraction in a published study [3]. In addition, we required that they did not have predicted signal sequences, nor were they found to be secreted in any of the studies used to define the secretome.

Clones received from PFGRC were sequenced to confirm their identity. They were moved into Gal4-DNA binding domain (DB) and Gal4-activation domain (AD) Y2H vectors using Gateway recombination (Invitrogen), and transformed into yeast strains Y8800 and Y8930 for Y2H interaction mapping, essentially as previously described [48,49]. In order to evaluate their performance in Y2H protein-protein interaction (PPI) mapping, we first tested the 399 Mtb ORFs expressed as DBs for pairwise interactions with 399 Mtb AD-fusions by testing individual DB clones with pools of ~100 AD clones. From the ~160,000 combinations queried, we found 22 PPI, representing 14 unique pairs (Table S1). Next, we screened for PPI between the Mtb secretome (339 ORFs) and controls (60 ORFS) as DB fusions with ~12,000 human ORFs as AD fusions, testing approximately 4 million interactions. Individual DB ORFs were mated to pools of 188 human AD ORFs. This identified 191 PPIs, exceeding the predicted stochastic false positive rate of the Y2H platform by nearly 6-fold [50]. Positive colonies were sequenced to identify the interacting pairs, and yeast were re-transformed with the corresponding plasmids to directly test pairwise interactions. Hits were those colonies that grew on media lacking histidine, indicating an interaction, and that failed to grow in the presence of cyclohexamide, which eliminates autoactivators. They were considered to repeat if they scored positively at least 3 out of 4 times on subsequent testing (Dataset S3).

**siRNAs**

siGENOME pools (Thermo Scientific) targeting Tsg101 (M-049922) and Rab7 (M-040859) were used at 30 nM for CFU experiments. The individual siRNAs in the Tsg101 pool were previously shown to give equivalent knock-down [51]. For Hrs, ON-TARGET*plus* pool was used for CFU experiments at 30nM, or individual ON-TARGET*plus* (J-055516-09 (#9) or J-055516-12 (#12)) siRNAs were used at 50 nM for trafficking experiments. Hrs ON-TARGET*plus* siRNA (#12) was a direct match to the human mRNA and was used at 50 nM in A549 cells. siGENOME Non-Targeting siRNA #1 and ON-TARGET*plus* Non-Targeting siRNA #1 (Thermo Scientific) were controls. Transfections were verified using siGLO RISC-Free siRNA (Thermo Scientific) and/or *Silencer*^TM^ KIF11 (Ambion).

**Plasmids**

Published plasmids were used for tetracycline-regulated-GFP [52] or constitutively-expressed GFP in BCG [53] and Mtb [54]. Mtb secretome ORFs were provided by Pathogen Functional Genomics Resource Center (http://pfgrc.jcvi.org/). Human Hrs was obtained from the Center for Cancer Systems Biology Human ORFeome v5.1 collection ([55,56] and <http://horfdb.dfci.harvard.edu/>) and used for expression in yeast. For mammalian Hrs expression, a myc-tagged construct was used [57]. To generate C-terminal tagged EsxG_Mt_-6xHis and EsxH_Mt_-FLAG for expression in mammalian cells, EsxG and EsxH were amplified by PCR from Gateway entry clones provided by PFGRC using the following primers: EsxG, forward: 5’-GGGAAGCTTATGAGCCTTTTGGATGCT-3’; EsxG, reverse: 5’-GGGGAATTCTCAATGATGATGGTGGTGGTGGAACCCGGTATAGGTCGA-3’; EsxH, forward: 5’-GGGAAGCTTATGTCGCAAATCATGTACAAC-3’; EsxH, reverse: 5’-CCCGAATTCTCACTTATCGTCGTCATCCTTGTAATCGCCGCCCCATTT-3’. They were cloned into the HindIII and EcoRI sites of pcDNA3.

To generate EsxG_Mt_ EsxH_Mt_-FLAG and EsxG_Ms_ EsxH_Ms_-FLAG for overexpression in mycobacteria, these regions were amplified from BCG (the EsxG-EsxH region is 100% identical between BCG and Mtb) and Msmeg genomic DNA, respectively. The primers used for EsxG_Mt_ EsxH_Mt_-FLAG were: forward: 5'-GCCATATGAGCCTTTTGGATGCT -3'; reverse: 5’-CCAAGCTTCTACTTGTCGTCGTCGTCCTTGTAGTCGCCGCCCCATTT-3’. The primers used for EsxG_Ms_ EsxH_Ms_-FLAG were: forward: 5’-GCCATATGAGTCTTCTCGACGCTCACAT-3’; reverse: 5’- CGAAGCTTCTACTTGTCGTCGTCGTCCTTGTAGTCTCCCCACTTGG-3’. The reverse primer encodes an in frame FLAG tag. The PCR products were cloned into NdeI and HindIII sites of pSYMP [58].

For expression in yeast and mammalian cells, *M. smegmatis* EsxH (MSMEG_0621) was amplified by PCR using primers containing attB1 and attB2 sites (forward: 5’-GGGGACAACTTTGTACAAAAAAGCAGGCTTCATGTCCCAGATCATGTAC-3’; reverse: 5’-GGGGACAACTTTGTACAAGAAAGCTGGGTCTCCCCACTTGGCGC-3’) to clone into pDONR221 using a Gateway BP reaction. It was subcloned into Y2H DB and AD expression vectors. Similarly, *M. smegmatis* EsxG (MSMEG_0620) was amplified by PCR using primers containing attB1 and attB2 sites and 5’ and 3’ gene sequence to clone into pDONR221 using a Gateway BP reaction. To generate C-terminal tagged EsxG_Ms_-6xHis and EsxH_Ms_-FLAG for expression in mammalian cells, they were PCR amplified from the Gateway entry clones using the following primers: EsxG, forward: 5’- CCCAAGCTTATGAGTCTTCTCGACGCTCACATC3’; EsxG, reverse: 5’- CCCGAATTCTCAATGATGATGGTGGTGGTGGATACCGGTGTAGGTGCTGGCCGC-3’; EsxH, forward: 5’-GCAAGCTTATGTCCCAGATCATGTACAACTACCCGG-3’; EsxH, reverse: 5’-CCCGAATTCTCACTTATCGTCGTCATCCTTGTAATCTCCCCACTTGGCGCCTTA-3’). They were cloned into the HindIII and EcoRI sites of pcDNA3.

The published EsxG-H fusion construct [59] was used to make recombinant EsxG_Mt_ EsxH_Mt_ heterodimer. The original construct encodes EsxG_Mt_ and EsxH_Mt_ as a single polypeptide, separated by a linker (GLVPRGSTG) containing a thrombin cleavage site. It lacks the first five amino acids of EsxH_Mt_, which we added using the QuikChange Lightening Site-Directed Mutagenesis kit (Agilent). This was used for production of recombinant protein.

The EsxG_Mt_-EsxH_Mt_ fusion protein was moved into pDONR221 using the Gateway BP reaction with the following primers (forward: 5’-GGGGACAACTTTGTACAAAAAAGTTGGCACCATGAGCCTTTTGGATGC-3’; reverse: 5’-GGGGACAACTTTGTACAAGAAAGTTGGCAAGCCGCCCCATTTGG-3’). A Gateway LR reaction was then carried out to subclone into the Y2H DB expression vector.

Murine and zebrafish Hrs constructs were cloned from murine cDNA (gift from S. Feske) and zebrafish cDNA (gift from H. Knaut) using primers containing attB1 and attB2 sites and 5’ and 3’ gene sequence (murine Hrs forward: 5’- GGGGACAACTTTGTACAAAAAAGTTGGCACCATGGGGCGAGGCAGC-3’; reverse: 5’-GGGGACAACTTTGTACAAGAAAGTTGGTCAGTCAAAGGAGATGAGC-3’; zebrafish Hrs forward: 5’- GGGGACAACTTTGTACAAAAAAGTTGGCACCATGGGCAAAGGCGG-3’; reverse: 5’-GGGGACAACTTTGTACAAGAAAGTTGGTCAGTCAAATGAAATGAGC-3’). A BP reaction was used to clone them into pDONR221, and they were subcloned into the Y2H AD destination vector. Two distinct zebrafish isoforms were cloned that behaved identically in Y2H assays. Hrs truncation mutants were made by PCR amplification from the full-length pDONR221 plasmid using the Gateway BP reaction. All constructs were sequenced; they were moved by Gateway cloning into yeast AD expression vectors for use in the Y2H and the C-terminal fragment (amino acids 398-777) was moved into pDEST40 for use in co-immunoprecipitations experiments.

**Recombinant protein purification**

*E.coli* (BL21(DE3); Invitrogen) transformed with the His-tagged EsxG_Mt_-EsxH_Mt_ [59] expression plasmid was grown in LB at 37ºC, induced with 450 μM Isopropyl β-D-1-thiogalactopyranoside (Promega) for 3h, harvested by centrifugation, and frozen at -80ºC. Pellets were resuspended in lysis buffer (300 mM NaCl, 50 mM NaH_2_PO_4_, Halt Protease Inhibitor cocktail) with 10 mM imidazole (Fisher BioReagents), 3 U/mL Benzonase Nuclease (Novagen), and 1 mg/ml of lysozyme (Sigma-Aldrich) at pH 8.0, incubated at room temperature (RT) for 40 min, and centrifuged at 10,000 X g for 30 min at 4ºC. Supernatant was incubated with Ni-NTA Superflow resin (Qiagen) at 4ºC for 1.5h. Resin was washed with lysis buffer and eluted in lysis buffer containing 250 mM imidazole. Thrombin (Novagen) was added (1 unit/ml) overnight at RT to cleave the linker connecting EsxG and EsxH. Samples were repurified on Ni-NTA. SDS-PAGE and Coomassie blue staining assessed purity and degree of thrombin cleavage. Proteins were quantified with a NanoDrop 1000 and/or by comparison to BSA standards on a Coomassie gel. Recombinant Hrs was prepared as described [60].

**Hill plot analysis**

To determine the dissociation constant and stoichiometry of Hrs- EsxG_Mt_-EsxH_Mt_ binding, the experimental data was fitted to the Hill equation, y= xη (Kd η+ xη) where Kd and η are the dissociation constant and the Hill coefficient, respectively. In the experiment, we measured the steady-state binding kinetics of Hrs to the EsxG_Mt_ EsxH_Mt_ heterodimer, using the fixed concentration (50 µM) of EsxG_Mt_ EsxH_Mt_ and increasing concentrations of Hrs (0 ~ 36.3 µM). At each data point, we calculated the free (unbound) Hrs concentration and the normalized (fraction) of Hrs-bound EsxG_Mt_ EsxH_Mt_ to which the Hill equation was fitted. However, under current experimental conditions, we may not be able to accurately measure the bound fraction with 100% efficiency, and the system may or may not be at steady-state equilibrium. For this reason, we also took into account this unknown “efficiency” (as an unknown parameter) during the curve optimization. The least square method, or the absolute difference between the empirical data and the Hill function, was used to obtain the optimized values for Kd and η. We also used the weight function to take into account the errors in the data (i.e., the standard error of the mean shown as the error bar in the figure). The optimized value is Kd= ~5.4 µM and η = 1.7. All numerical analyses were carried out under the MATLAB environment (The MathWorks, Natick, MA).

**Hrs and FK2 staining**

For Hrs and FK2 immunostaining, antibodies used were: Hrs (Santa Cruz Biotechnology) and FK2 (Millipore). Cells were permeabilized with 0.05% saponin-PBS prior to fixation in 3% paraformaldehyde in PBS. They were incubated with primary antibody overnight at 4°C, washed and incubated with anti-rabbit Alexa-594 for Hrs and anti-mouse Alexa-488 for FK2.

**Supporting References**

1. de Souza GA, Malen H, Softeland T, Saelensminde G, Prasad S, et al. (2008) High accuracy mass spectrometry analysis as a tool to verify and improve gene annotation using Mycobacterium tuberculosis as an example. BMC Genomics 9: 316.

2. Malen H, Berven FS, Fladmark KE, Wiker HG (2007) Comprehensive analysis of exported proteins from Mycobacterium tuberculosis H37Rv. Proteomics 7: 1702-1718.

3. Rosenkrands I, King A, Weldingh K, Moniatte M, Moertz E, et al. (2000) Towards the proteome of Mycobacterium tuberculosis. Electrophoresis 21: 3740-3756.

4. Champion PA, Stanley SA, Champion MM, Brown EJ, Cox JS (2006) C-terminal signal sequence promotes virulence factor secretion in Mycobacterium tuberculosis. Science 313: 1632-1636.

5. Fortune SM, Jaeger A, Sarracino DA, Chase MR, Sassetti CM, et al. (2005) Mutually dependent secretion of proteins required for mycobacterial virulence. Proc Natl Acad Sci U S A 102: 10676-10681.

6. Mattow J, Schaible UE, Schmidt F, Hagens K, Siejak F, et al. (2003) Comparative proteome analysis of culture supernatant proteins from virulent Mycobacterium tuberculosis H37Rv and attenuated M. bovis BCG Copenhagen. Electrophoresis 24: 3405-3420.

7. Garces A, Atmakuri K, Chase MR, Woodworth JS, Krastins B, et al. (2010) EspA acts as a critical mediator of ESX1-dependent virulence in Mycobacterium tuberculosis by affecting bacterial cell wall integrity. PLoS Pathog 6: e1000957.

8. Bahk YY, Kim SA, Kim JS, Euh HJ, Bai GH, et al. (2004) Antigens secreted from Mycobacterium tuberculosis: identification by proteomics approach and test for diagnostic marker. Proteomics 4: 3299-3307.

9. Dave JA, Gey van Pittius NC, Beyers AD, Ehlers MR, Brown GD (2002) Mycosin-1, a subtilisin-like serine protease of Mycobacterium tuberculosis, is cell wall-associated and expressed during infection of macrophages. BMC Microbiol 2: 30.

10. Målen H, Søfteland T, Wiker HG (2008) Antigen analysis of Mycobacterium tuberculosis H37Rv culture filtrate proteins. Scand J Immunol 67: 245-252.

11. Raghavan S, Manzanillo P, Chan K, Dovey C, Cox JS (2008) Secreted transcription factor controls Mycobacterium tuberculosis virulence. Nature 454: 717-721.

12. Weldingh K, Rosenkrands I, Jacobsen S, Rasmussen PB, Elhay MJ, et al. (1998) Two-dimensional electrophoresis for analysis of Mycobacterium tuberculosis culture filtrate and purification and characterization of six novel proteins. Infect Immun 66: 3492-3500.

13. Gomez M, Johnson S, Gennaro ML (2000) Identification of secreted proteins of Mycobacterium tuberculosis by a bioinformatic approach. Infect Immun 68: 2323-2327.

14. Saint-Joanis B, Demangel C, Jackson M, Brodin P, Marsollier L, et al. (2006) Inactivation of Rv2525c, a substrate of the twin arginine translocation (Tat) system of Mycobacterium tuberculosis, increases beta-lactam susceptibility and virulence. J Bacteriol 188: 6669-6679.

15. McDonough JA, Hacker KE, Flores AR, Pavelka MS, Jr., Braunstein M (2005) The twin-arginine translocation pathway of Mycobacterium smegmatis is functional and required for the export of mycobacterial beta-lactamases. J Bacteriol 187: 7667-7679.

16. Braunstein M, Brown AM, Kurtz S, Jacobs WR (2001) Two nonredundant SecA homologues function in mycobacteria. J Bacteriol 183: 6979-6990.

17. Braunstein M, Espinosa BJ, Chan J, Belisle JT, Jacobs WR (2003) SecA2 functions in the secretion of superoxide dismutase A and in the virulence of Mycobacterium tuberculosis. Mol Microbiol 48: 453-464.

18. Wiker HG, Wilson MA, Schoolnik GK (2000) Extracytoplasmic proteins of Mycobacterium tuberculosis - mature secreted proteins often start with aspartic acid and proline. Microbiology 146 ( Pt 7): 1525-1533.

19. Chubb AJ, Woodman ZL, da Silva Tatley FM, Hoffmann HJ, Scholle RR, et al. (1998) Identification of Mycobacterium tuberculosis signal sequences that direct the export of a leaderless beta-lactamase gene product in Escherichia coli. Microbiology 144 ( Pt 6): 1619-1629.

20. Bach H, Papavinasasundaram KG, Wong D, Hmama Z, Av-Gay Y (2008) Mycobacterium tuberculosis virulence is mediated by PtpA dephosphorylation of human vacuolar protein sorting 33B. Cell Host Microbe 3: 316-322.

21. Deghmane AE, Soualhine H, Soulhine H, Bach H, Sendide K, et al. (2007) Lipoamide dehydrogenase mediates retention of coronin-1 on BCG vacuoles, leading to arrest in phagosome maturation. J Cell Sci 120: 2796-2806.

22. Master SS, Rampini SK, Davis AS, Keller C, Ehlers S, et al. (2008) Mycobacterium tuberculosis prevents inflammasome activation. Cell Host Microbe 3: 224-232.

23. Walburger A, Koul A, Ferrari G, Nguyen L, Prescianotto-Baschong C, et al. (2004) Protein kinase G from pathogenic mycobacteria promotes survival within macrophages. Science 304: 1800-1804.

24. Sun J, Wang X, Lau A, Liao TY, Bucci C, et al. (2010) Mycobacterial nucleoside diphosphate kinase blocks phagosome maturation in murine RAW 264.7 macrophages. PLoS ONE 5: e8769.

25. Samuel LP, Song CH, Wei J, Roberts EA, Dahl JL, et al. (2007) Expression, production and release of the Eis protein by Mycobacterium tuberculosis during infection of macrophages and its effect on cytokine secretion. Microbiology 153: 529-540.

26. Skeiky YA, Lodes MJ, Guderian JA, Mohamath R, Bement T, et al. (1999) Cloning, expression, and immunological evaluation of two putative secreted serine protease antigens of Mycobacterium tuberculosis. Infect Immun 67: 3998-4007.

27. Rengarajan J, Murphy E, Park A, Krone CL, Hett EC, et al. (2008) Mycobacterium tuberculosis Rv2224c modulates innate immune responses. Proc Natl Acad Sci U S A 105: 264-269.

28. Xu J, Laine O, Masciocchi M, Manoranjan J, Smith J, et al. (2007) A unique Mycobacterium ESX-1 protein co-secretes with CFP-10/ESAT-6 and is necessary for inhibiting phagosome maturation. Mol Microbiol 66: 787-800.

29. Hett EC, Chao MC, Steyn AJ, Fortune SM, Deng LL, et al. (2007) A partner for the resuscitation-promoting factors of Mycobacterium tuberculosis. Mol Microbiol 66: 658-668.

30. Abdallah AM, Verboom T, Hannes F, Safi M, Strong M, et al. (2006) A specific secretion system mediates PPE41 transport in pathogenic mycobacteria. Mol Microbiol 62: 667-679.

31. Li Y, Miltner E, Wu M, Petrofsky M, Bermudez LE (2005) A Mycobacterium avium PPE gene is associated with the ability of the bacterium to grow in macrophages and virulence in mice. Cell Microbiol 7: 539-548.

32. Ribeiro-Guimarães ML, Pessolani MC (2007) Comparative genomics of mycobacterial proteases. Microb Pathog 43: 173-178.

33. Chopra P, Koduri H, Singh R, Koul A, Ghildiyal M, et al. (2004) Nucleoside diphosphate kinase of Mycobacterium tuberculosis acts as GTPase-activating protein for Rho-GTPases. FEBS Lett 571: 212-216.

34. McLaughlin B, Chon JS, MacGurn JA, Carlsson F, Cheng TL, et al. (2007) A mycobacterium ESX-1-secreted virulence factor with unique requirements for export. PLoS Pathog 3: e105.

35. Lodes MJ, Dillon DC, Mohamath R, Day CH, Benson DR, et al. (2001) Serological expression cloning and immunological evaluation of MTB48, a novel Mycobacterium tuberculosis antigen. J Clin Microbiol 39: 2485-2493.

36. Cascioferro A, Delogu G, Colone M, Sali M, Stringaro A, et al. (2007) PE is a functional domain responsible for protein translocation and localization on mycobacterial cell wall. Mol Microbiol 66: 1536-1547.

37. Koul A, Choidas A, Treder M, Tyagi AK, Drlica K, et al. (2000) Cloning and characterization of secretory tyrosine phosphatases of Mycobacterium tuberculosis. J Bacteriol 182: 5425-5432.

38. Raynaud C, Guilhot C, Rauzier J, Bordat Y, Pelicic V, et al. (2002) Phospholipases C are involved in the virulence of Mycobacterium tuberculosis. Mol Microbiol 45: 203-217.

39. Målen H, Berven FS, Søfteland T, Arntzen M, D'Santos CS, et al. (2008) Membrane and membrane-associated proteins in Triton X-114 extracts of Mycobacterium bovis BCG identified using a combination of gel-based and gel-free fractionation strategies. Proteomics 8: 1859-1870.

40. Sassetti CM, Boyd DH, Rubin EJ (2003) Genes required for mycobacterial growth defined by high density mutagenesis. Mol Microbiol 48: 77-84.

41. Siegrist MS, Unnikrishnan M, McConnell MJ, Borowsky M, Cheng TY, et al. (2009) Mycobacterial Esx-3 is required for mycobactin-mediated iron acquisition. Proc Natl Acad Sci U S A 106: 18792-18797.

42. Serafini A, Boldrin F, Palù G, Manganelli R (2009) Characterization of a Mycobacterium tuberculosis ESX-3 conditional mutant: essentiality and rescue by iron and zinc. J Bacteriol 191: 6340-6344.

43. Rohde KH, Abramovitch RB, Russell DG (2007) Mycobacterium tuberculosis invasion of macrophages: linking bacterial gene expression to environmental cues. Cell Host Microbe 2: 352-364.

44. Sassetti CM, Rubin EJ (2003) Genetic requirements for mycobacterial survival during infection. Proc Natl Acad Sci U S A 100: 12989-12994.

45. Rengarajan J, Bloom BR, Rubin EJ (2005) Genome-wide requirements for Mycobacterium tuberculosis adaptation and survival in macrophages. Proc Natl Acad Sci U S A 102: 8327-8332.

46. Pethe K, Swenson DL, Alonso S, Anderson J, Wang C, et al. (2004) Isolation of Mycobacterium tuberculosis mutants defective in the arrest of phagosome maturation. Proc Natl Acad Sci U S A 101: 13642-13647.

47. Stewart GR, Patel J, Robertson BD, Rae A, Young DB (2005) Mycobacterial mutants with defective control of phagosomal acidification. PLoS Pathog 1: 269-278.

48. Rual JF, Venkatesan K, Hao T, Hirozane-Kishikawa T, Dricot A, et al. (2005) Towards a proteome-scale map of the human protein-protein interaction network. Nature 437: 1173-1178.

49. Dreze M, Monachello D, Lurin C, Cusick ME, Hill DE, et al. (2010) High-quality binary interactome mapping. Methods Enzymol 470: 281-315.

50. Venkatesan K, Rual JF, Vazquez A, Stelzl U, Lemmens I, et al. (2009) An empirical framework for binary interactome mapping. Nat Methods 6: 83-90.

51. Philips JA, Porto MC, Wang H, Rubin EJ, Perrimon N (2008) ESCRT factors restrict mycobacterial growth. Proc Natl Acad Sci U S A 105: 3070-3075.

52. Ehrt S, Guo XV, Hickey CM, Ryou M, Monteleone M, et al. (2005) Controlling gene expression in mycobacteria with anhydrotetracycline and Tet repressor. Nucleic Acids Res 33: e21.

53. Teitelbaum R, Cammer M, Maitland ML, Freitag NE, Condeelis J, et al. (1999) Mycobacterial infection of macrophages results in membrane-permeable phagosomes. Proc Natl Acad Sci U S A 96: 15190-15195.

54. Wolf AJ, Desvignes L, Linas B, Banaiee N, Tamura T, et al. (2008) Initiation of the adaptive immune response to Mycobacterium tuberculosis depends on antigen production in the local lymph node, not the lungs. J Exp Med 205: 105-115.

55. Lamesch P, Li N, Milstein S, Fan C, Hao T, et al. (2007) hORFeome v3.1: a resource of human open reading frames representing over 10,000 human genes. Genomics 89: 307-315.

56. Rual JF, Hirozane-Kishikawa T, Hao T, Bertin N, Li S, et al. (2004) Human ORFeome version 1.1: a platform for reverse proteomics. Genome Res 14: 2128-2135.

57. Haag J, Chubinskaya S, Aigner T (2006) Hgs physically interacts with Smad5 and attenuates BMP signaling. Exp Cell Res 312: 1153-1163.

58. Festa RA, McAllister F, Pearce MJ, Mintseris J, Burns KE, et al. (2010) Prokaryotic ubiquitin-like protein (Pup) proteome of Mycobacterium tuberculosis [corrected] . PLoS One 5: e8589.

59. Callahan B, Nguyen K, Collins A, Valdes K, Caplow M, et al. (2010) Conservation of structure and protein-protein interactions mediated by the secreted mycobacterial proteins EsxA, EsxB, and EspA. J Bacteriol 192: 326-335.

60. Tsujimoto S, Bean AJ (2000) Distinct protein domains are responsible for the interaction of Hrs-2 with SNAP-25. The role of Hrs-2 in 7 S complex formation. J Biol Chem 275: 2938-2942.

61. Via LE, Fratti RA, McFalone M, Pagan-Ramos E, Deretic D, et al. (1998) Effects of cytokines on mycobacterial phagosome maturation. J Cell Sci 111 ( Pt 7): 897-905.

**Supplementary Figure Legends**

Figure S1. siRNA-mediated depletion of Hrs and Rab7.

(A) RAW264.7 (RAW) cells were treated with 50nM ON-TARGET*plus* individual siRNAs (#9 or #12) targeting Hrs or control for 2 d. (B) RAW cells were treated with increasing concentration of siRNA#9 targeting Hrs for 2 or 5 days. (C) A549 cells treated with 50 nM Hrs siRNAs (#12) or control for 2d. (A)–(C) Western blotting with antibody recognizing Hrs was used to assess silencing. (D) RAW cells were treated with 30 nM siRNA targeting Rab7 or control. Silencing was assessed 2d later by western blotting using an antibody recognizing Rab7. (E) RAW cells treated with control siRNA (siCON) or siRNA targeting Hrs (#9 or #12) for 2d were examined by immunofluorescence using antibodies against Hrs, shown in red, and ubiquitinated proteins (FK2) in green.

Figure S2. siRNAs targeting Hrs and Rab7 enhance the intracellular survival of BCG in BMDMs.

4 x 10^4^ BMDMs were transfected with 30 nM siRNA pools targeting Hrs (ON-TARGET*plus*) or Rab7 (siGENOME) 6-8d after harvest. 3d later, they were infected with BCG (MOI of 2 to 5). CFU were enumerated 2 days post-infection and are normalized to the average number of CFU in control wells from two independent experiments. Results reflect the mean +/- SEM. **p*<0.05; ***p*<0.01, unpaired Student’s *t*-test**.**

Figure S3. Automated Image Analysis of Phagosome Maturation

(A) For quantifying the degree of co-localization between bacteria and cellular markers or Lysotracker, images were background subtracted and analyzed using the Binary Operation Analysis within NIS Elements Software. Bacteria were selected in the green channel. The region the software has selected that corresponds to the bacteria is shown in red in the second panel. That region was expanded (dilate binary) and then eroded and a binary operation was performed to generate a “donut” in the region surrounding the bacteria. The region of interest (ROI) is shown in purple. The mean fluorescence intensity (MFI) in the ROI was determined for the cellular marker. Bacteria were analyzed from at least three fields per sample per experiment. We confirmed that automated quantification closely paralleled manual quantification and visual scoring by a blinded observer.

(B) To further validate the automated analysis, we verified enhanced LAMP1 co-localization in macrophages pre-treated with IFN-γ, which has been shown to promote mycobacterial phagosome maturation [61]. Mtb-GFP was used to infect RAW cells treated with control siRNA (siCON) and either pre-treated with IFN-γ or solvent control 24 hours prior to infection. In IFN-γ pre-tretated macrophages there is a significant shift in LAMP1 co-localization around bacterial phagosomes. Data points are the MFI of LAMP1 around bacteria 24 hpi; bars show mean +/- SEM; *p* <0.0001.

(C) Co-localization of Lamp1, Lysotracker, and TfR with metabolically active BCG compared to co-localization with total BCG. RAW cells were treated with control siRNA (siCON) and infected with BCG constitutively expressing GFP (BCG-GFP) or BCG expressing GFP under a tetracycline inducible promoter (BCG-tet-GFP). AnTc was added 24 hpi to induce expression of GFP. Because it takes > 12h for the strain to become detectably GFP positive, co-localization between BCG-tet-GFP and LAMP1, LysoTracker or TfR was measured at 48 hpi. For the BCG-GFP strain, LAMP1 and TfR were examined at 48 hpi and LysoTracker at 24 hpi. Data points are the MFI around bacteria; bars show mean +/- SEM; *p* value of BCG-tet-GFP compared to BCG-GFP for LAMP1=0.0081, for LysoTracker <0.0001, for TfR=0.0046.

Figure S4. Quantification of EsxH_Mt_–FLAG in transfected HEK293 cells.

EsxH_Mt_ was co-transfected with vector control, EsxG_Mt_, or Hrs as indicated. Prior to protein harvest, cells were treated with DMSO or MG132. EsxH_Mt_-FLAG levels were quantified from at least three independent experiments using ImageJ software. **p*<0.05; ***p*<0.01, unpaired Student’s *t*-test; ns- not significant**.** Whiskers reflect the minimum and maximum data points, while the cross bars show the median.

Figure S5. Treatment with MG132 does not result in higher molecular weight forms of the EsxH proteins.

HEK293 cells were transfected with plasmids as indicated. Cells were either treated with DMSO or MG132 prior to protein harvest. Lysates were examined for mono- and polyubiquitinated proteins using the FK2 antibody. The EsxH proteins were visualized using the FLAG antibody. No differences were seen in the mobility of EsxH_Mt_, EsxH_Ms_, or EsxH_Mt_-H76AE77A in the presence of MG132.

Figure S6. EsxG_Mt_ EsxH_Mt_-FLAG is not secreted by Msmeg.

Msmeg transformed with empty vector, EsxG_Mt_ EsxH_Mt_-FLAG, or EsxG_Ms_ EsxH_Ms_-FLAG were analyzed for the presence of EsxH in the pellet and culture filtrate (CF). dlaT (Rv2215), a cytosolic protein, was used as a loading control and to indicate the degree of bacterial lysis.

Figure S7. EsxG_Mt_ EsxH_Mt_-FLAG does not alter intracellular growth of Mtb.

RAW cells were infected with Mtb containing vector control, EsxG_Mt_ EsxH_Mt_-FLAG, or EsxG_Ms_ EsxH_Ms_-FLAG and bacterial CFU were enumerated at 3h, 24h and 48h post-infection. No statistically significant differences were seen at any time point. Results reflect the mean +/- SEM.
